# Lumbar cerebrospinal fluid-to-brain extracellular fluid surrogacy is context-specific: insights from LeiCNS-PK3.0 simulations

**DOI:** 10.1007/s10928-021-09768-7

**Published:** 2021-06-17

**Authors:** Mohammed A. A. Saleh, Chi Fong Loo, Jeroen Elassaiss-Schaap, Elizabeth C. M. De Lange

**Affiliations:** 1 Division of Systems Biomedicine and Pharmacology, Leiden Academic Center for Drug Research, Leiden University, Leiden, The Netherlands; 2 PD-Value B.V, Houten, The Netherlands

**Keywords:** Physiologically-based pharmacokinetic models, CNS, Drug development, Brain

## Abstract

Predicting brain pharmacokinetics is critical for central nervous system (CNS) drug development yet difficult due to ethical restrictions of human brain sampling. CNS pharmacokinetic (PK) profiles are often altered in CNS diseases due to disease-specific pathophysiology. We previously published a comprehensive CNS physiologically-based PK (PBPK) model that predicted the PK profiles of small drugs at brain and cerebrospinal fluid compartments. Here, we improved this model with brain non-specific binding and pH effect on drug ionization and passive transport. We refer to this improved model as Leiden CNS PBPK predictor V3.0 (LeiCNS-PK3.0). LeiCNS-PK3.0 predicted the unbound drug concentrations of brain ECF and CSF compartments in rats and humans with less than two-fold error. We then applied LeiCNS-PK3.0 to study the effect of altered cerebrospinal fluid (CSF) dynamics, CSF volume and flow, on brain extracellular fluid (ECF) pharmacokinetics. The effect of altered CSF dynamics was simulated using LeiCNS-PK3.0 for six drugs and the resulting drug exposure at brain ECF and lumbar CSF were compared. Simulation results showed that altered CSF dynamics changed the CSF PK profiles, but not the brain ECF profiles, irrespective of the drug’s physicochemical properties. Our analysis supports the notion that lumbar CSF drug concentration is not an accurate surrogate of brain ECF, particularly in CNS diseases. Systems approaches account for multiple levels of CNS complexity and are better suited to predict brain PK.

**Supplementary Information:**

The online version contains supplementary material available at 10.1007/s10928-021-09768-7.

## Introduction

Central nervous system (CNS) pharmacokinetic (PK) profiling, though challenging, remains critical for drug development. Two PK profiles can be distinguished in the CNS: brain and cerebrospinal fluid (CSF) PK profiles. In CNS drug development, compounds are selected that optimize brain PK profile, since brain cells and extracellular fluid (ECF) represent the major site of drug (side-) effects. Suboptimal drug exposure in brain has resulted in clinical trial failure and has ultimately contributed to the high attrition rate of the CNS drugs in development [1]. CSF represents a relatively accessible matrix to sample the CNS, mainly via lumbar puncturing. While lumbar CSF drug concentrations predict brain concentrations better than that of plasma [2], its accuracy as a surrogate of brain PK has been argued [3], particularly for low passive permeability and actively transported drugs [4].

The major challenge in designing drugs with adequate brain PK, is the poor understanding of the role of CNS (patho)physiology in determining brain PK [5]. Up to this challenge, a mechanistic, systems-based understanding of key physiological and pathological processes in healthy and diseased CNS is instrumental in predicting brain (patho-) pharmacokinetics.

Our group previously published a comprehensive CNS physiologically-based (PBPK) model that predicts the unbound concentration–time profiles of small drugs within the CNS [6, 7]. This model, hereafter referred to as Leiden CNS PBPK predictor 1.0 (LeiCNS-PK1.0), was developed using knowledge-based, bottom-up modeling [6, 7], without using in vivo-measured PK profiles for model building. The mechanistic structure of LeiCNS-PK1.0 allows interspecies and interpopulation translation and provides a framework to study the effect of altering a single or multiple physiological aspects on CNS PK. Thus, LeiCNS-PK1.0 can be used to predict mechanistically the effect of disease-altered CNS physiology on unbound drug exposure in brain [7]. While LeiCNS-PK1.0 could adequately predict the CNS PK profiles of rats and healthy humans [6, 7], several components of CNS physiology, including brain tissue non-specific binding and pH impact on passive transport, were represented in a rudimentary manner. This limited the translatability of LeiCNS-PK1.0 predictions between species and from healthy to diseased populations. First, the calculated pH factors did not reflect the neutral drug fraction of a given compartment, as neutral drug fraction in each compartment was normalized to that of the plasma compartment. In addition, it was assumed that the charged drug molecules do not undergo transcellular or paracellular passive transport across the blood–brain (BBB) and blood-cerebrospinal fluid (BCSFB) barriers, which is not physiologically plausible as charged drugs can be transported via the passive paracellular route [8]. Accounting for the impact of pH on drug ionization has been shown to clearly improve the prediction of CNS PK profiles of drugs with weak acidic and/or basic groups [9]. Drug non-specific binding, on the other hand, lacked a mechanistic description and physiological plausibility as it was assumed in LeiCNS-PK1.0 to occur instantaneously within the ECF and was calculated using the unbound drug fraction in brain and plasma, brain tissue composition, and lipophilicity of the drug. Brain unbound drug fraction (f_u,b_) as measured in vitro, varies between measurement techniques, requires brain tissue, and might not be available at early stages of drug development. Brain non-specific binding has been demonstrated to be one of the major determinants of brain pharmacokinetics [10], particularly for lipophilic drugs [11–14]. Hence, LeiCNS-PK1.0 required improvement.

In this paper, we first improve LeiCNS-PK1.0 by readdressing the effect of pH on drug ionization, LeiCNS-PK1.0 assumptions related to passive transport of charged molecules at BBB and BCSFB barriers, and the time-dependent brain tissue non-specific binding. We refer to this improved model as Leiden CNS PBPK predictor 3.0 or LeiCNS-PK3.0. Next, we use LeiCNS-PK3.0 model to explore the effect of altered CSF dynamics on CSF and brain ECF PK profiles as well as on predictability of brain ECF drug concentration by that of lumbar CSF. Changes in CSF dynamics, CSF volume and flow, are common in CNS diseases (Table 1) and often alter CSF PK; their effect on the brain ECF PK profiles remains unexplored [15].

**Table 1:** Cerebrospinal fluid dynamics in different CNS disease conditionsa

	Aging^a^	Alzheimer's disease	Hydrocephalus	Traumatic brain injury^a^
CSF volume	400%	150%^b^	150%^b^	115%
	[51]	[52]	[53]	[54]
CSF production	66%	46%^a^	60%^b^	
	[55]	[56]	[57]	
CSF flow	150%	Normal CSF flow^a^	370% and reverse flow direction^a^	
	[58]	[59]	[32]	
CSF clearance	Reduced CSF absorption^c^	65%^b^	20–60%^a^	
	[60]	[61]	[62]	

^a^Compared to adults (< 60 years)

^b^Compared to elderly (60 + years)

^c^A study in rats

## Methods

### CNS and plasma in vivo-measured drug concentrations

Drugs used to validate the model predictions included acetaminophen, atenolol, methotrexate, morphine, phenytoin, raclopride, risperidone, paliperidone, remoxipride, quinidine, oxycodone, and indomethacin. These drugs were selected to cover the physicochemical space of small drug molecules with molecular weights between 100 and 500 g/mol, different ionization rate constants and charge class at physiological pH, different lipophilicity, and different affinity to active transporters at the BBB and BCSFB.

Plasma PK data, for the development of the empirical plasma models, and CNS PK data, for the evaluation of LeiCNS-PK3.0 predictions, were available for both rats and humans from the literature. Supplementary table 1 summarizes the sampling location and data references.

For validating the rat version of LeiCNS-PK3.0, only in-house data were used, where individual unbound PK profiles were simultaneously measured in the same animal under controlled conditions in plasma and in multiple CNS locations: brain ECF, lateral ventricles (LV), and cisterna magna (CM) using microdialysis, in addition to total brain concentrations, which were measured with the brain homogenate method. Clinical brain PK profiles measured with microdialysis are quite rare due to ethical restrictions. In humans, individual unbound PK profiles of brain ECF and lumbar CSF were available from patients with conditions that do not affect CNS physiology or from healthy, uninjured sites. Acetaminophen and indomethacin concentrations were measured in patients with nerve root compression. Oxycodone were available from patients undergoing elective gynecological surgery. Morphine concentrations were collected using microdialysis from uninjured brain tissue sites from traumatic brain injury patients.

Total drug concentrations were corrected using respective fraction of unbound drug where needed. CSF drug concentrations were assumed unbound due to the low protein content of the CSF, i.e. f_u,CSF_ = 1, except for indomethacin with an f_u,CSF_ of 0.47 [16].

### Drug-specific parameters

Drug specific parameters: lipophilicity (logP_o/w_), acid/base ionization constants (pK_a_/pK_b_), and molecular weight, were collected from Drugbank [17] and are listed in Table 2. Calculated logP_o/w_ values by ALOGPS method [18] were used, unless experimental logP_o/w_ values were available, while calculated pK_a_/pK_b_ values by the MARVIN method provided by CHEMAXON [19] were used.

**Table 2:** Drug-specific parameters

Drug	Molecular weight	Charge class	pK_a_	pK_b_	logP	Kp_uu,ECF_	AF_in,ECF_	AF_out,ECF_	Kp_uu,LV_	AF_in,LV_	AF_out,LV_	Kp_uu,CM_	AF_in,CM/SAS_	AF_out,CM/SAS_
Acetaminophen	151.16	Neutral	9.46	− 4.4	0.46	0.51^a^	1	12.5	0.51^a^	1	86.8	0.51^a^	1	90
						1^b^	1	1	1^b^	1	1	1^b^	1	1
Atenolol	266.34	Base	14.08	9.67	0.16	0.037^a^	1	9.9E^4^	0.037^a^	1	8.3E^5^	0.037^a^	1	8.3E^5^
Indomethacin	357.8	Acid	3.79	− 2.9	4.27	0.1^c^	1	5.7	0.272^g^	1	47.3	0.272^g^	1	48.1
Methotrexate	454.45	Acid	3.41	2.81	− 1.85	0.018^a^	1	4.1E^8^	0.0066^a^	1	9.5E^9^	0.0024^a^	1	2.6E^10^
Morphine	285.34	Base	10.26	9.12	0.87	0.38^a, d^	1	372.8	0.38^a, d^	1	2999.5	0.38^a, d^	1	3088.1
						0.23^a, e^	1	764.5	0.23^a, e^	1	6248	0.23^a, e^	1	6336.6
						0.23^f^	1	170.4	0.23^f^	1	671.8	0.23^f^	1	677.2
Oxycodone	315.37	Base	13.56	8.21	0.7	1.69^f^	17.6	1	2^g^	100.6	1	2^g^	94.7	1
Paliperidone	426.49	Base	13.74	8.76	1.8	0.5^a^	1	13.7	0.5^a^	1	90.8	0.5^a^	1	96
Phenytoin	252.27	Neutral	9.47	− 9	2.47	0.26^a^	1	4.2	0.26^a^	1	6.5	0.26^a^	1	6.5
Quinidine	324.42	Base	13.89	9.05	3.44	1.5^a^	1.5	1	1.5^a^	3.9	1	1.5^a^	3.6	1
Raclopride	347.24	Amphoteric	5.31	9.32	3.38	1.1^a^	16	1	1.1^a^	187.2	1	1.1^a^	120.5	1
Remoxipride	371.28	Base	13.06	8.4	2.1	0.8^a^	1	2.2	0.8^a^	1	6.2	0.8^a^	1	7.5
Risperidone	410.49	Base	NA	8.76	3.27	0.97^a^	1	1.3	0.97^a^	1	1.2	0.97^a^	1	1.4

^a^Values are measured in rats

^b^Assumed value based on physiological knowledge, used for human predictions[7]

^c^Values are measured in rats and corrected for human translation as described in [7]; indomethacin is MRP4 [63], MRP6 [64], OAT1[65], OAT3 [66] substrate. Expression levels of these transporters are at least eightfold lower in humans than in rats [67–69]. No information on interspecies differences of transporter activity could be identified and was then assumed the same. Thus, AF factors were calculated using rat values and then divided by scaling factor = 8

^d^Dose: 4 mg/kg

^e^Dose: 10, 40 mg/kg

^f^Values are measured in rats and corrected for human translation as described in[7]

^g^Values measured in humans

### Leiden CNS PBPK predictor V3.0 (LeiCNS-PK3.0)

#### Model development

**Fig. 1:**
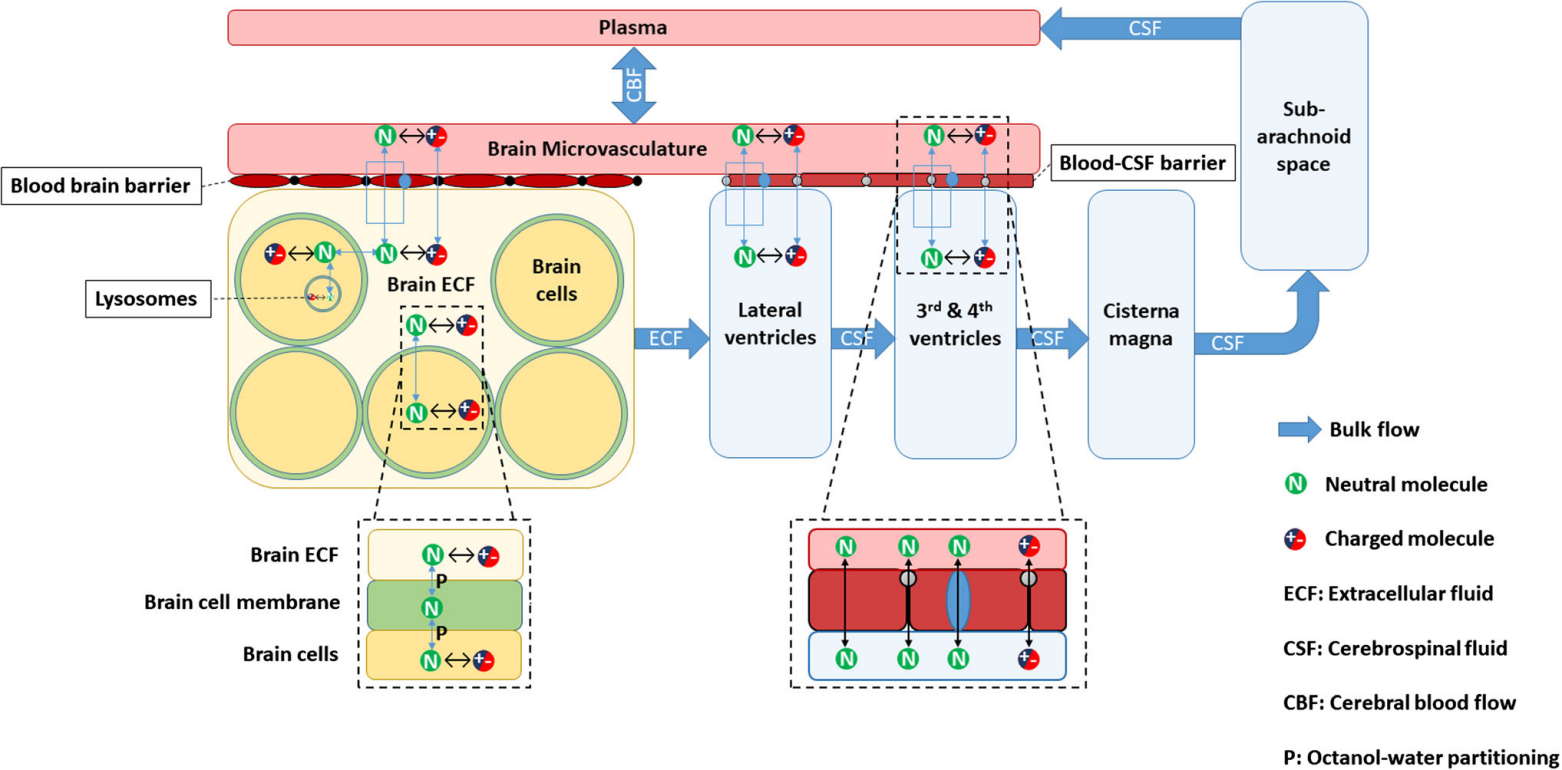
LeiCNS-PK3.0 model structure. LeiCNS-PK3.0 is composed of a whole body empirical plasma model and a CNS PBPK model. Both models are connected via cerebral blood flow

LeiCNS-PK3.0 (Fig. 1 and Supplementary Fig. 1) consists of an empirical plasma model, which predicts plasma PK, and a nine-compartment CNS model. The empirical plasma model serves as an input that drives the PK of the CNS model, with both models linked by the cerebral blood flow. Development of the empirical plasma model and detailed description of the CNS model structure, physiological processes, and transport modes are described below. The physiological parameters of rats and humans are presented in Supplementary table 2. When multiple values were found in the literature, the mean value was used.

LeiCNS-PK3.0 is an improvement of the published LeiCNS-PK1.0 [6, 7] on aspects related to brain non-specific binding, pH effect on drug ionization, and assumptions related to transcellular and paracellular passive diffusion of the charged drug molecules. A comparison of the improved aspects in LeiCNS-PK3.0 compared to LeiCNS-PK1.0 is presented in Table 3.

**Table 3:** Comparison of the improved aspects in LeiCNS-PK3.0 versus LeiCN-PK1.0

Aspect	LeiCNS-PK1.0	LeiCNS-PK3.0
pH factor (PHF)	Defined as the ratio of the neutral fraction of a drug of a given compartment to that of plasma	Defined as the neutral fraction of a drug in a given compartment
	Calculated using Henderson-Hasselbalch equations with pH of the compartment, pH of plasma, and the drug-specific ionization constant	Calculated using adapted Henderson-Hasselbalch equations using compartment specific pH and the drug-specific ionization constant
Brain tissue non-specific binding	Using binding factor	Mechanistic description
	Instantaneous	According to diffusion clearance between aqueous and lipid phases
	Binding occurs within the brain ECF to a hypothetical compartment	Binding occurs to the phospholipids of the brain cell membrane
	Relies on total brain-to-plasma concentration ratio (K_p_). K_p_ is calculated using drug lipophilicity (logP), unbound drug fraction in brain (f_u,b_) and plasma (f_u,p_), and brain and plasma tissue composition	Relies on drug lipophilicity and the volume of brain phospholipids
Passive paracellular transport	Paracellular route is restricted to neutral drug only	Paracellular route is available for both neutral and charged drug

#### CNS compartments

In LeiCNS-PK3.0, different CNS compartments are accounted for: brain microvessels, brain extracellular fluid (ECF), brain intracellular fluid (ICF), lysosomes, cranial cerebrospinal fluid (CSF) compartments: lateral ventricles, third and fourth ventricles, and cisterna magna, in addition to the CSF in subarachnoid space (SAS), including lumbar CSF. A new compartment, brain cell membrane, has been added to LeiCNS-PK3.0, as the assumed non-specific binding site in brain.

#### pH effect on drug ionization

The pH factors (PHF) are defined as the neutral fraction of the drug concentration of a given compartment. PHF is determined using adapted Henderson-Hasselbalch equations utilizing compartment-specific pH (pH_comp_) and the ionization constants of the strongest acidic group (pk_a_) and the strongest basic group (pk_b_) of the drug. In case of drugs missing one group (e.g. risperidone has only a basic group, but no acidic groups), the relevant neutral fraction of this missing group is set to 1. PHF is calculated as per the equations below.

$$\text{Neutral\; fraction\; of\; acidic\; group}\; \left({PHF}_{acidic}\right) = \frac{1}{1+{10}^{{pH}_{comp} -{ pk}_{a}}}$$

$$\text{Neutral\; fraction\; of\; basic\; group}\; \left({PHF}_{basic}\right) = \frac{1}{1+{10}^{{pk}_{b} - {pH}_{comp}}}$$

$$\text{Neutral\; fraction\; of \;drug}\; \left(PHF\right)={PHF}_{acidic} \times {PHF}_{basic}$$

#### Brain tissue non-specific binding

In LeiCNS-PK3.0, brain phospholipids, which constitute a major fraction of brain cell membranes, are assumed as the non-specific binding site in brain [20–22]. The volume of the brain cell membrane compartment is 5% of the total brain volume, which represents the volume fraction of phospholipids in the brains of rats [23] and humans [24]. CL_wo_ and CL_ow_ (mL min^−1^) describe the diffusion clearance of a given drug between brain ECF and ICF on one side and brain cell membrane on the other side. At steady state, the ratio of the drug concentration in the brain cell membrane to the drug concentration in the brain ECF and ICF is equal to the octanol–water partition coefficient (P_oct-water_).

#### Bulk fluid flow

Bulk fluid flow refers to the drug clearance between CNS compartments due to fluid flow, irrespective of the concentration gradients. In LeiCNS-PK3.0, bulk flows include cerebral blood flow between the brain microvessels and the central compartment of the empirical plasma model, ECF bulk flow from brain ECF to LV, and the CSF flow from the cranial CSF to the absorption sites in SAS.

#### Passive transport

Passive transport in the CNS involves paracellular and transcellular transport. Transcellular transport refers to the permeability of the drug through phospholipid bilayer of the membranes of the BBB endothelial cells, BCSFB epithelial cells, brain parenchyma, and lysosomes. Paracellular transport describes the aqueous diffusion of the drug molecules between the cells of the BBB and BCSFB via the openings of the tight junctions. Further details on the equations required to calculate aqueous diffusion and transmembrane permeability are reported in the supplementary information and in [6].

In LeiCNS-PK3.0, neutral drug molecules are transported through both transcellular and paracellular routes, whereas charged drug molecules are transported via paracellular routes only. Anions, cations, and zwitterions are assumed to undergo paracellular diffusion at the same rate.

#### Asymmetry factors

In LeiCNS-PK3.0, physiological processes that are not explicitly addressed such as active transport across the BBB and BCSFB, and metabolism, are accounted for using asymmetry factors (AF). AF were calculated using the LeiCNS-PK3.0 equations at steady state and Kp_uu_, the ratio of the unbound drug concentration in a given tissue to that of plasma. Kp_uu_ values were available from the literature or calculated using influx and efflux clearances of a given compartment [25].

$${Kp}_{uu}=\frac{{Cl}_{in}}{{Cl}_{out}}$$

where Kp_uu_ is the ratio of unbound concentration of a given tissue compartment to that of plasma at steady state, Cl_in_ is the total influx clearance into the tissue compartment, and Cl_out_ total efflux clearance out of the tissue compartment. Influx and efflux clearances can be estimated using available unbound drug concentration–time profiles. In humans, Kp_uu_ values are not often available and can be calculated as described in the decision tree presented in [7]. If in vivo-measured Kp_uu_ values are unavailable, AF can be derived from in vitro estimates such as efflux ratio and cell uptake values as we described previously [7, 26].

Equations for calculating AF are provided in the supplementary materials. Influx AF (AF_in_) and efflux AF (AF_ef_) are calculated at BBB, BCSFB_LV_, and BCSFB_TFV_, where three scenarios are possible depending on the value of Kp_uu_. Kp_uu_ equal to 1 suggests an equilibrium of drug concentration across BBB/BCSFB, and thus AF_in_ and AF_ef_ are equal to 1. Kp_uu_ smaller than 1 suggests active efflux at BBB/BCSFB; in this case AF_in_ is set to 1, while AF_ef_ is calculated using the relevant equation and the associated Kp_uu_ value. Kp_uu_ larger than 1 suggests active influx at BBB/BCSFB, AF_ef_ is set to 1, and then AF_in_ is calculated [7].

The calculated AF values are listed in Table 2. The AF factors of atenolol and methotrexate were exceptionally high, which can be attributed mainly to their very low Kp_uu_ values. Atenolol (Kp_uu_ = 0.037) is a low passive permeability molecule and recent evidence show that atenolol might undergo active transport at the BBB [27]. Methotrexate (Kp_uu_ = 0.018, 0.0066, 0.0024 for ECF, LV, and CM, respectively) is a substrate of PGP [28], BCRP [29], and MRP4 [30], which are three main transporters at the BBB and BCSFB. At CNS physiological pH, methotrexate acts as an anion, whose negative charge could reduce its passive permeability as a result of the interaction with negatively charged phospholipids of the cell membranes. The combined low passive permeability and presence of active transport contribute to the low Kp_uu_ of both drugs.

### Empirical plasma PK models

Rat plasma PK models were developed using non-linear mixed effects modeling, where one-, two-, three- compartment models were compared. Interindividual variability was tested using an exponential model for every PK parameter. Residual unexplained variability was included using either proportional or combined proportional/additive error models.

The final model was selected based on likelihood ratio test with p < 0.05, equivalent to a decrease of the objective function value of 3.84; visual predictive check (VPC) plots to compare the model fit to drug concentrations in plasma; precision of the parameter estimates denoted by the %relative standard errors; and the basic goodness of fit plots that include individual/population predictions versus observations and conditional weighted residuals versus population prediction/time.

Human plasma PK models were either available from the literature or developed in a similar fashion as described for rats.

### LeiCNS-PK3.0 evaluation

LeiCNS-PK3.0 model performance was evaluated using visual prediction check plots (VPCs), where the median and 95% prediction interval of 200 model simulations were plotted against and compared to in vivo-measured unbound drug concentrations. The model simulations accounted for interindividual variability and residual variabilities of the plasma PK model, as described above. The relevant η of interindividual variability and ε of residual unexplained variabilities were randomly sampled from a normal distribution with a mean of 0 and a variance of ω^2^ and σ^2^, respectively, and transformed as required.

Next, prediction errors were calculated using the individual measured drug concentrations and their corresponding time-matched simulations median. Average fold error (AFE) was calculated to evaluate the model’s bias, while absolute average fold error (AAFE) was calculated to compare the typical PK profile simulated by the model to the typical PK profile of the measured PK data. A typical profile is the profile predicted assuming no interindividual variability, i.e. when etas are set to zero. AFE and AAFE were calculated using relative accuracy calculated for each drug. AFE and AAFE values approaching 100% denote accurate model predictions.

Relative Accuracy of a given drug (RA_drug_) at a given compartment was calculated as follows:

$$RA_{{drug}} = \frac{1}{M}\sum\limits_{{i = 1}}^{N} {\sum\limits_{{j = 1}}^{m} {\log _{{10}} \left( {\frac{{MedP_{{i,j}} }}{{Obs_{{i,j}} }}} \right)} }$$

$$M=\sum\limits_{i=1}^{N}m$$

where Obs_i,j_ is jth observation of the ith individual; MedP_i,j_ is the median value of the 200 simulations corresponding to Obs_i,j_; M is the total number of observations of all individuals; m is the number of observations of the ith individual; and N is the total number of individuals.

%AFE of a given compartment was calculated as:

$$AFE=\frac{1}{D}\sum\limits_{d=1}^{D}{RA}_{drug}$$

$$\% AFE = 100 \times 10^{{AFE}}$$

where D is the number of drugs used for evaluation.

%AAFE of a given compartment was calculated as:

$$AAFE=\frac{1}{D}\sum\limits_{d=1}^{D}\left|{RA}_{drug}\right|$$

$$\% AAFE = 100\; \times 10^{{AAFE}}$$

In addition, the mean absolute relative accuracy (MARA) was calculated to evaluate the variability of individual drug concentrations around the median of LeiCNS-PK3.0 simulations within a given compartment. MARA was based on absolute relative accuracy of a given drug (ARA_drug_) at a given compartment, which was calculated as:

$${ARA}_{drug}=\frac{1}{M}\sum\limits_{i=1}^{N} \sum\limits _{j=1}^{m}\left|{log}_{10}\left(\frac{{MedP}_{i,j}}{{Obs}_{i,j}}\right)\right|$$

$$MARA=\frac{1}{D}\sum\limits_{d=1}^{D}{ARA}_{drug}$$

$$\%MARA=100 \times {10}^{MARA}$$

where Obs_i,j_ is jth observation of the ith individual; MedP_i,j_ is the median value of the 200 simulations corresponding to Obs_i,j_; M is the total number of observations of all individuals; m is the number of observations of the ith individual; N is the total number of individuals; and D is the number of drugs used for evaluation.

Symmetric mean absolute prediction errors (SMAPE) were calculated to benchmark LeiCNS-PK3.0 with LeiCNS-PK1.0. A SMAPE value closer to 0% implies a more accurate model.

$$SMAPE\left( \% \right) = \frac{{100}}{M}\sum\limits_{{i = 1}}^{N} {\sum\limits_{{j = 1}}^{m} {\left| {\frac{{2*\left( {Obs_{{i,j}} - MedP_{{i,j}} } \right)}}{{Obs_{{i,j}} + MedP_{{i,j}} }}} \right|} }$$

where Obs_i,j_ is jth observation of the ith individual; MedP_i,j_ is the median value of the 200 simulations corresponding to Obs_i,j_; M is the total number of observations of all individuals; m is the number of observations of the ith individual; and N is the total number of individuals.

### The effect of altered CSF dynamics on brain ECF PK

The effect of altered CSF volume and flow on the drug exposure in the brain ECF and CSF was studied using human LeiCNS-PK3.0. Simulations were performed for six drugs with different physicochemical properties. Test drugs included methotrexate, acetaminophen, phenytoin, atenolol, raclopride, and risperidone. A fixed 1-compartment plasma PK model of human was applied across all drugs in order to isolate the impact of CSF parameters from other variables. Rat Kp_uu_ values and the associated AF were adapted for humans. The resulting drug concentration ratio of brain ECF-to-SAS was compared between the physiological, two- and five-fold CSF volume and flow. SAS in this setting represents lumbar CSF PK profile, while brain ECF represents the brain PK profile, assuming no active transport takes place at the level of the brain cells. Brain ECF is an intermediate compartment between brain microvasculature and brain cells and therefore unaltered drug exposure in brain ECF will imply unaltered drug exposure in brain cells. Two- and five-folds changes were selected to reflect the changes of CSF volume and CSF flow in CNS diseases as reported in Table 1. For example, the volume of the ventricles increase by 4.57%/year during healthy aging [31], which in the course of 20 years will result in the expansion of the ventricles to about 250%. The CSF flow, measured at the aqueduct of patients with idiopathic normal pressure hydrocephalus patients, increases to 370% of its physiological value [32].

### Sensitivity analysis

A sensitivity analysis was performed using the human version of LeiCNS-PK3.0 to identify the main parameters that define the PK profiles at the brain ECF, brain ICF and SAS. The sensitivity analysis was carried out using four drugs with distinct physicochemical properties: acetaminophen, morphine, methotrexate, and raclopride. The CNS parameters were varied individually by 1.1, 1.5, and 2 folds, and resulting PK descriptors, C_max_, T_max_, and AUC, in the selected compartments were compared to those of the physiological situation, using the sensitivity index calculated as:

$$Sensitivity\, index={log}_{2}\frac{{Y}_{d}}{{Y}_{o}}$$

where Y_d_ and Y_o_ are the pharmacokinetic descriptors (C_max_, T_max_, and AUC) of the altered and physiological values, respectively.

### Data analysis and software

Plasma PK model parameters were estimated using NONMEM version 7.4.3 (ICON, Dublin, Ireland) [33]. General data analysis and visualization and LeiCNS-PK3.0 simulations were performed using R version 3.6.1 [34], where simulations were performed using RxODE package version 0.9.1-0 [35], using the LSODA (Livermore Solver for Ordinary Differential Equations) Fortran package. Algebraic equations were solved using Maxima Computer Algebra System version 19.01.2x (available from http:// maxima.sourceforge.net). Literature data were extracted with WebPlotDigitizer version 4.2 (https://apps.automeris.io/wpd/).

## Results

### Plasma PK models

The empirical plasma model parameters of the rat and human are displayed in Table 4. Rat plasma PK model parameters were estimated with good precision and the models accurately described the observed plasma drug concentrations. The plasma PK model of methotrexate, however, slightly overpredicted the data. Human plasma models of acetaminophen and morphine were available from the literature [36], while plasma PK model parameters of oxycodone and indomethacin were developed.

**Table 4:** Rat and human empirical plasma PK models used as input to LeiCNS-PK3.0 model

	Plasma PK parameter estimates						Interindividual variability						Residual variability	
	Cl_cen_ (mL min^−1^)	Q_cen-per1_ (mL min^−1^)	Q_cen-per2_ (mL min^−1^)	V_cen_ (mL)	V_per1_ (mL)	V_per2_ (mL)	Cl_cen_ (%)	Q_cen-per1_ (%)	Q_cen-per2_ (%)	V_cen_ (%)	V_per1_ (%)	V_per2_ (%)	Proportional (%)	Additive (ng mL^−1^)
Rat plasma PK models														
Acetaminophen	4.70	11.18	31.35	50.71	27,891.70	162.47	35.5	0.0	0.0	0.0	0.0	0.0	26.7	0.0
Atenolol	6.30	4.25	0.0	118.70	203.36	0.0	8.9	0.0	0.0	0.0	0.0	0.0	14.7	0.0
Methotrexate	8.31	23.33	0.79	38.16	139.95	47.99	36.5	0.0	52.3	0.0	0.0	29.5	17.6	0.0
Morphine	23.34	4.97	31.68	175.73	1636.28	475.86	46.9	93.9	0.0	92.5	0.0	49.2	24.2	0.0
Paliperidone	219.46	6765.63	0.0	25.00	32,981.00	0.0	44.4	0.0	0.0	0.0	45.5	0.0	20.0	0.0
Phenytoin	47.72	415.88	0.0	453.32	2268.39	0.0	65.8	0.0	0.0	122.5	22.0	0.0	16.3	1571.0
Quinidine	178.28	238.03	753.99	183.65	7335.00	5062.54	26.1	38.4	0.0	0.0	0.0	0.0	23.4	20.8
Raclopride	45.40	68.04	15.03	50.44	468.42	690.00	13.6	0.0	0.0	0.0	0.0	0.0	14.3	0.0
Remoxipride	47.43	16.52	56.76	82.89	602.77	457.52	31.0	0.0	29.8	124.4	36.4	44.7	21.3	0.0
Risperidone	773.30	0.0	0.0	47,936.80	0.0	0.0	89.9	0.0	0.0	66.9	0.0	0.0	29.6	12.9
Human plasma PK models														
Acetaminophen	495.00	0.0	0.0	108,000.00	0.0	0.0	0.0	0.0	0.0	0.0	0.0	0.0	23.9	0.0
Indomethacin^a^	14,200.00	54,600.00	0.0	1,320,000.00	10,300,000.00	0.0	0.0	145.9	0.0	0.0	0.0	0.0	22.3	0.0
Morphine	3070.00	3030.00	0.0	16,000.00	95,400.00	0.0	27.1	0.0	0.0	59.6	0.0	0.0	9.6	0.0
Oxycodone	1140.00	11,700.00	47.40	93,600.00	178,000.00	19,400.00	31.1	0.0	0.0	86.7	0.0	0.0	19.1	0.0

^a^Intramuscular dosing was used with bioavailability of 100% [70] and estimated absorption rate constant of 2,850,000 (min^−1^)

### Model evaluation

The CNS model of LeiCNS-PK3.0 was developed using bottom-up modeling relying on physiological information only. Evaluation of the model predictions was performed using published PK data from different brain regions, and thus model evaluation is independent from model development.

#### Rat LeiCNS-PK3.0 evaluation

**Fig. 2:**
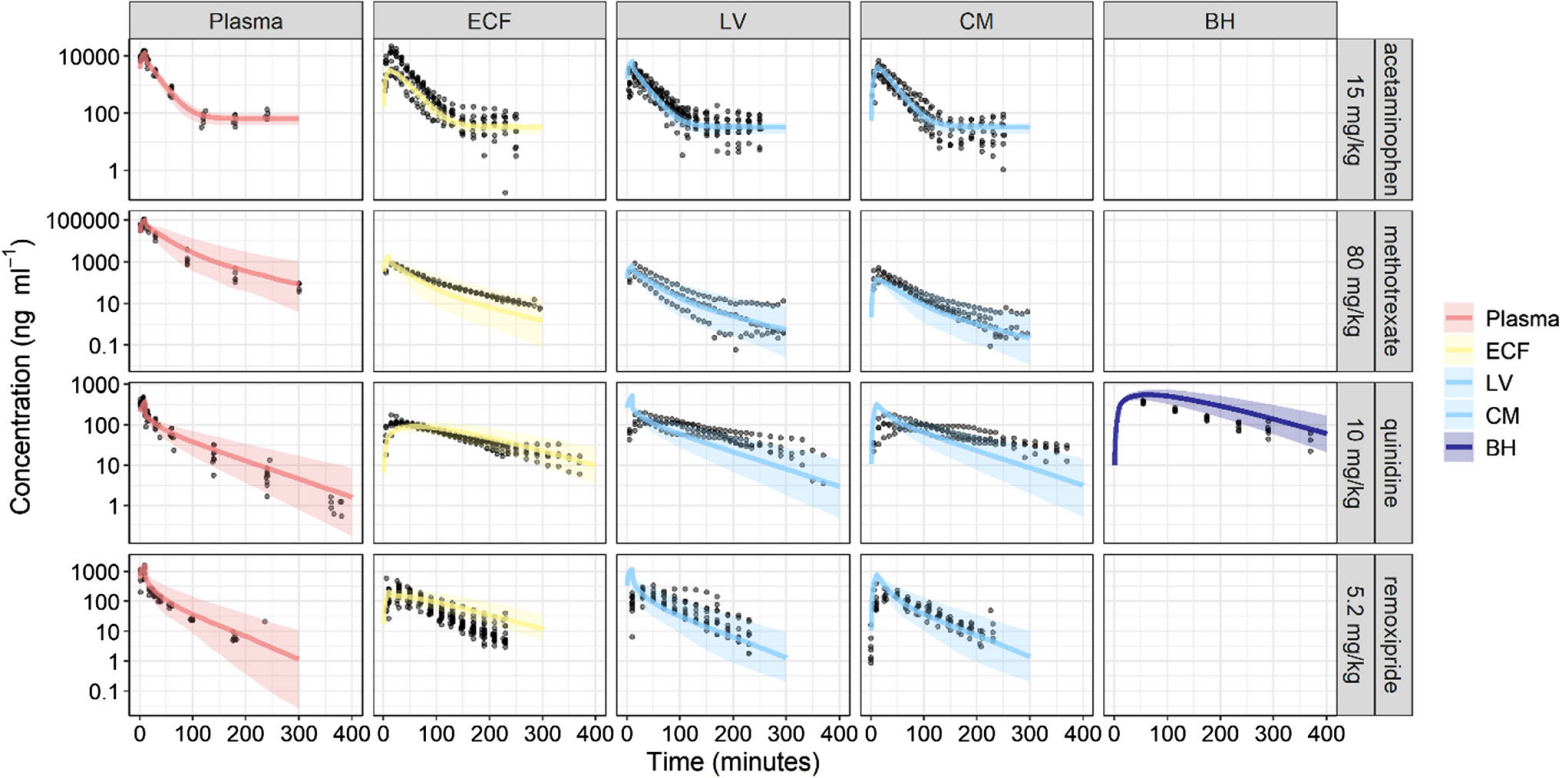
Model evaluation of the rat LeiCNS-PK3.0 model. Visual predictive checks plots compared in vivo measured drug concentration (black dots) in multiple CNS locations to the median (solid line) and 95% prediction intervals (colored band) of 200 model simulations. *ECF* brain extracellular fluid, *LV* lateral ventricles, *CM* cisterna magna, *BH* brain homogenate (Color figure online)

Figure 2 and Supplementary Fig. 2a-b depict the VPC plots of rat LeiCNS-PK3.0 simulations against the measured drug concentrations of 10 drugs (Supplementary table 1). LeiCNS-PK3.0 adequately predicted the observed data in the brain ECF, lateral ventricles (LV), and cisterna magna (CM), with some exceptions. Methotrexate brain ECF and quinidine 20 mg LV concentrations were slightly underpredicted. Phenytoin brain ECF and CM and quinidine CM concentrations were underpredicted towards the end of the simulation. Remoxipride 4, 8, 16 mg predictions captured the peak of the observations but overpredicted the remaining observations. LeiCNS-PK3.0 additionally predicted brain homogenate (BH) concentrations, but less adequately. The model overpredicted quinidine and remoxipride 0.7 mg and underpredicted phenytoin 40 mg observations and raclopride peak concentration.

In addition, LeiCNS-PK3.0 performance was evaluated by calculating the relative accuracy error and its derivatives: %AFE and %AAFE that assess the model’s bias and typical PK profile predictability, respectively. Supplementary Fig. 2C displays a box plot of relative accuracy errors. %AFE (95% confidence interval) of brain ECF, LV, CM and BH were 90% (67–120), 77% (41–146), 80% (56–116), and 64% (6–643), respectively. These values deviate by a maximum of 35% from the optimum value of 100% and are indeed within two-fold error. %AAFE (95% confidence interval) were 140% (118–167), 139% (85- 229), and 149% (120–185) for brain ECF, LV and CM, respectively, which deviate by < 50% and are within two-fold error. BH predictions were less accurate, with a %AAFE of 322% (99–1045).

SMAPEs, besides, were calculated for comparison with LeiCNS-PK1.0. SMAPE of LeiCNS-PK3.0 (vs LeiCNS-PK1.0) were 65% (vs 72%), 71% (vs 71%), 70% (vs 69%), and 105% (vs 91%) for brain ECF, LV and CM and BH, respectively.

#### Human LeiCNS-PK3.0 evaluation

**Fig. 3:**
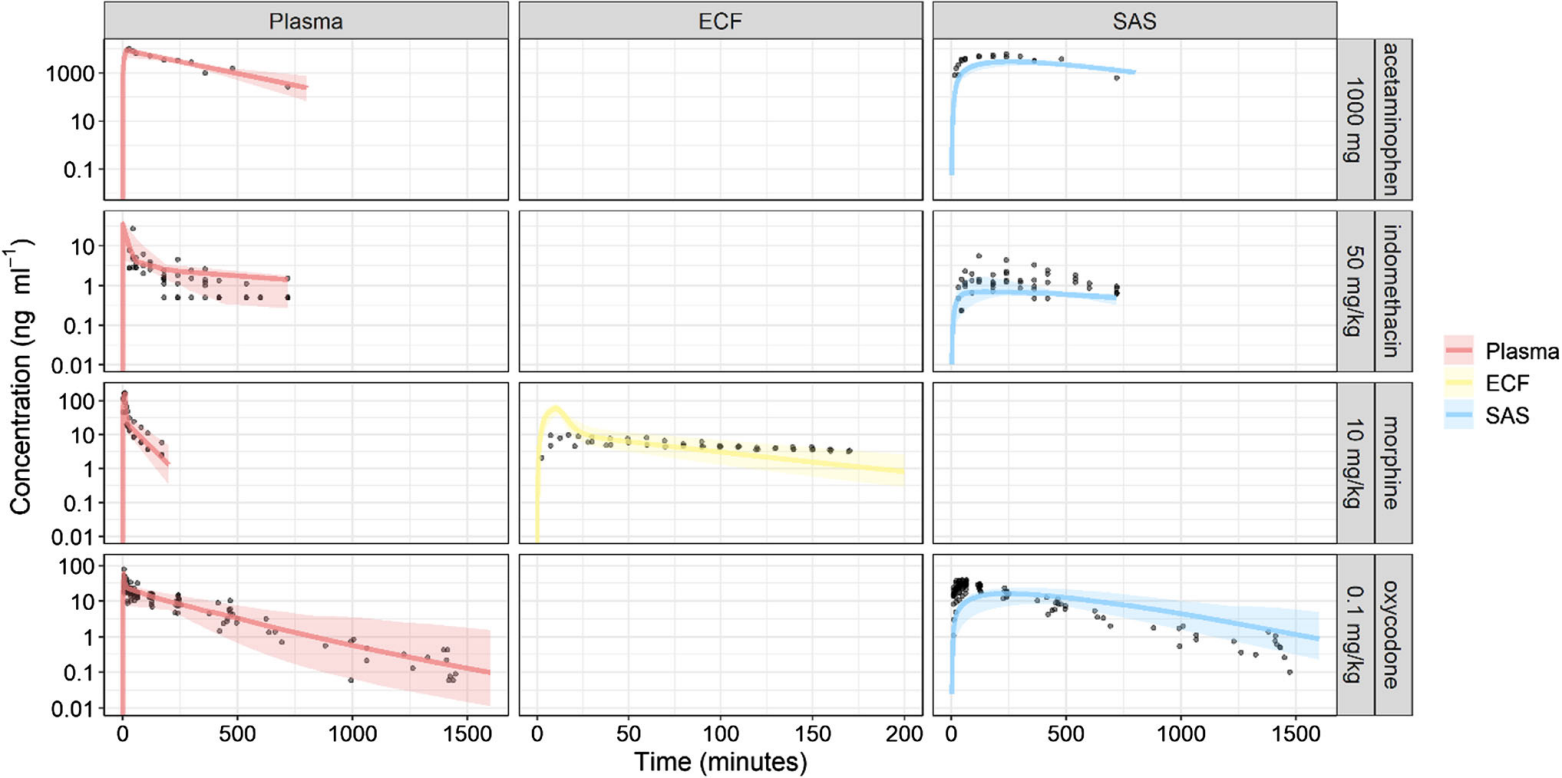
Model evaluation of the human LeiCNS-PK3.0 model. Visual predictive checks plots compared in vivo measured drug concentration (black dots) in multiple CNS locations to the median (solid line) and 95% prediction intervals (colored band band) of 200 model simulations. *ECF* brain extracellular fluid, *SAS* subarachnoid space (Color figure online)

Figure 3 displays the VPC plots of the human LeiCNS-PK3.0 simulations against the measured concentration–time profiles of four drugs (Supplementary table 1). The plots show that LeiCNS-PK3.0 adequately predicted the brain ECF and SAS concentrations. Acetaminophen and indomethacin SAS concentration were underpredicted to some extent. %AFE (Supplementary Fig. 3) of brain ECF and SAS were 92% and 56%, respectively. %AAFE of brain ECF and SAS were 109% and 179%, respectively. All error values were within the two-fold error limit.

### Effect of altered CSF dynamics on brain ECF and CSF pharmacokinetics

**Fig. 4:**
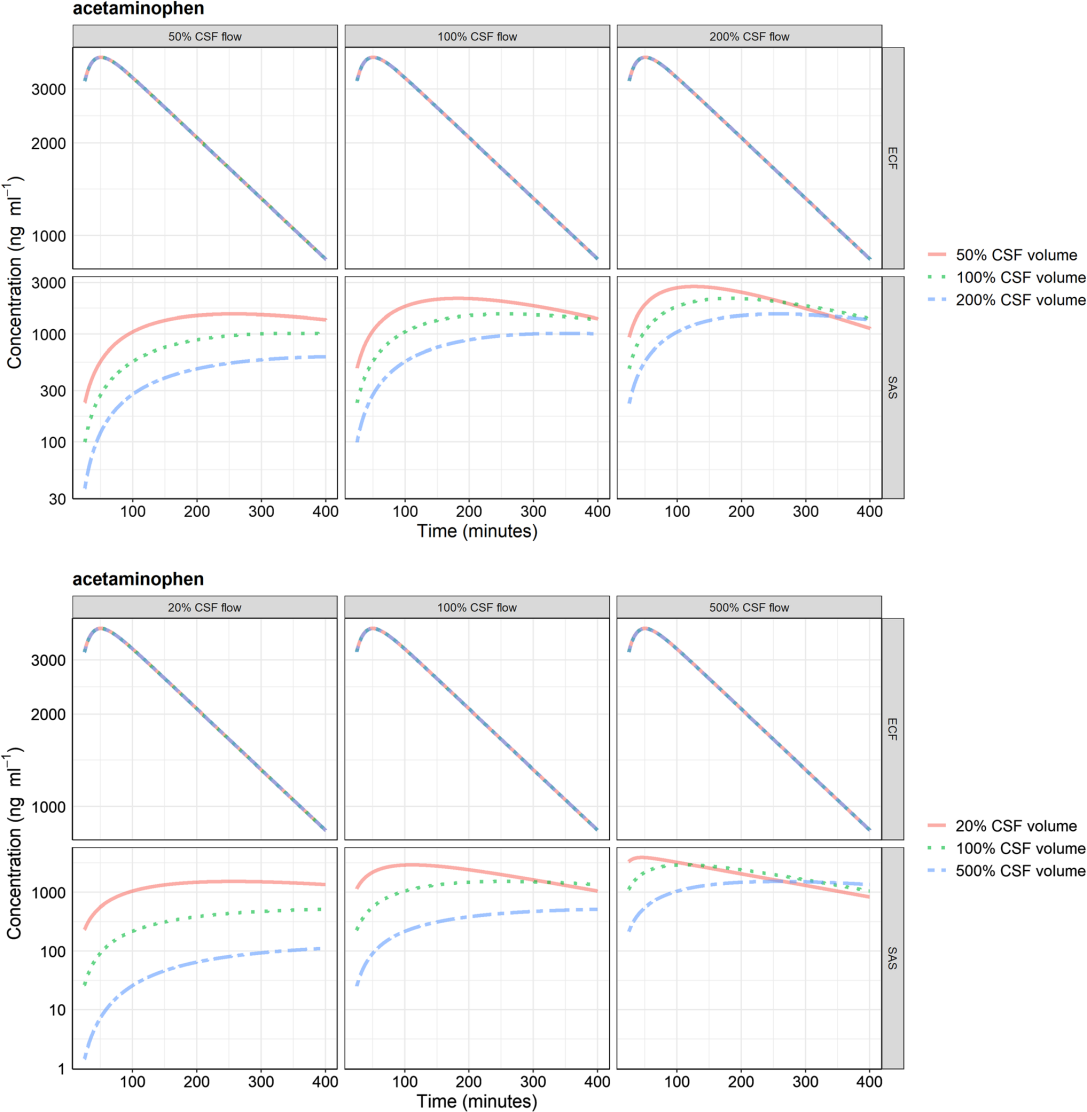
Pharmacokinetic profiles of acetaminophen at brain extracellular (ECF) fluid and subarachnoid space (SAS) at physiological and **a** two- and **b** five-fold altered cerebrospinal fluid (CSF) volume and flow. Changing CSF dynamics affects SAS pharmacokinetics and not brain ECF pharmacokinetics. *ECF* brain extracellular fluid, *SAS* subarachnoid space

PK profiles of brain ECF and SAS compartments at different CSF flow and volumes are shown in Fig. 4a, b for acetaminophen and Supplementary Fig. 4 a-e and 5 a-e for methotrexate, phenytoin, atenolol, raclopride, and risperidone. Changes in CSF volume and flow altered SAS but not brain ECF PK profile and hence changed the brain ECF-SAS ratio. Within the SAS, decrease in CSF volume or increase in CSF flow results in an earlier T_max_, higher C_max_, and a faster clearance. The observed changes of T_max_ and C_max_ at the SAS compartment was the same for all drugs regardless their physicochemical properties.

### Sensitivity analysis

LeiCNS-PK3.0 sensitivity analysis was performed to identify the CNS model parameters that influence the PK profiles at the brain ECF, brain ICF, and SAS. The identified parameters were drug- and CNS compartment-dependent. Brain ECF and ICF PK profiles were sensitive to active transport at BBB as reflected by brain-to-plasma unbound drug partitioning (Kp_uu,ECF_), volume and surface area of brain cells, width of BBB and tight junction pore, and pH of brain ECF and ICF. The SAS PK profile was sensitive to active transport at BCSFB given by the CSF-to-plasma unbound drug partitioning (Kp_uu,CM_), CSF flow, and SAS volume. LeiCNS-PK3.0 sensitivity analysis results are shown in Supplementary Fig. 6.

## Discussion

LeiCNS-PK3.0 simulations showed that altered CSF dynamics resulted in a shift in the drug concentration ratio of brain ECF-to-SAS CSF, where SAS CSF PK profiles but not brain ECF PK profiles were affected. This observation is independent of the drug’s physicochemical properties, as it is assumed in the model that transport into and out of the SAS CSF is mediated by CSF flow and does not involve barrier transport. This implies a context-specific surrogacy of lumbar CSF-to-brain ECF PK profiles and thus this relationship is not suitable for interpopulation or interspecies translation. LeiCNS-PK3.0 simulations, thus, reproach the classical assumption of the prediction of lumbar CSF drug concentration to brain ECF drug concentrations [2], which is in line with previous findings [3].

### LeiCNS-PK3.0 performance

LeiCNS-PK3.0 is an improved and a more mechanistic version of LeiCNS-PK1.0 [6, 7], where the physiological processes of non-specific binding and pH effect on drug ionization and passive transport across BBB and BCSFB have been addressed. LeiCNS-PK3.0 predictions are based exclusively on plasma PK, CNS physiological parameters, drug physicochemical properties, and in vitro measurements. LeiCNS-PK3.0 predicts brain non-specific binding using a drug property, i.e. lipophilicity, which is either measured at the early stages of drug development or predicted with QSAR approaches. This makes lipophilicity more efficient to use compared to the formerly-used brain unbound drug fraction (f_u,b_), which requires brain tissue.

LeiCNS-PK3.0 predictions are predominantly unbiased as indicated by the below 35% %AFE. The model, however, slightly underpredicts drug concentrations of human SAS, but within the two-fold error margin. Drug concentration–time profiles of rat brain ECF, LV, and CM and of human brain ECF and SAS were adequately predicted. %AAFE errors, which indicate the model prediction of typical PK profiles, were within the two-fold error limit, with human brain ECF predictions deviating less than 10%.

LeiCNS-PK3.0 predicted BH PK profiles less adequately which could be the result of the unaccounted for physiological processes such as brain metabolism, active transport at the brain cells, specific binding of drugs to target receptor, etc. BH predictions of raclopride, a known dopamine D2 receptor substrate [37], displayed the largest error among other drugs. %AAFE of BH without including raclopride was 223% compared to 322% with raclopride. Future inclusion of receptor binding and other physiological process is anticipated to improve LeiCNS-PK3.0 predictions.

LeiCNS-PK3.0 predictions of human brain intracellular fluid (ICF) PK profiles are depicted in supplementary Figs. 8. Both brain ECF and ICF represent the sites of drug action, which makes their PK profiles of top interest to drug developers. Brain ICF PK profiles cannot be validated with in vivo PK profiles, as such data are not attainable. Imaging techniques do not distinguish intracellular and extracellular drug. The brain slice method could be used to investigate the concentration and time dependency of the equilibrium between the brain ECF, represented by the buffer, and brain ICF [38]. This in vitro method is, however, limited by the loss of the whole brain context as a number of physiological processes such as bulk flows are missed, in addition to the limited duration of tissue viability.

### Sensitivity analysis: implications to LeiCNS-PK3.0 assumptions

A number of LeiCNS-PK3.0 parameters were calculated based on certain assumptions about CNS physiology, some of which were found by the sensitivity analysis to largely affect CNS PK. The affected assumptions were: surface of the brain cells membrane (SA_BCM_), CSF flow, and active transport.

SA_BCM_ was calculated using brain cells volume and number, assuming that all brain cells are spheres of equal radii. CSF flow was assumed constant in ventricles and the subarachnoid space, which does not reflect the physiology. Active transport was accounted for by calculating AF using Kp_uu_ whose value is dependent on dosing and measurement techniques. Improving the mechanistic description of these parameters should be a priority of future investigations and will increase the confidence in LeiCNS-PK3.0 predictions.

### New non-specific binding model

Brain non-specific binding in LeiCNS-PK3.0 is presented as a time-dependent process; a diffusion clearance describes the drug partitioning between brain ECF/ICF and phospholipids of the brain cell membrane. This is based on two assumptions. First, phospholipids of the brain cell membrane play a determinant role in non-specific binding within brain compared to the negligible role of brain proteins e.g. albumin [23, 39], neutral lipids [20], and other components of brain cells [22]. The second assumption relates to P_oct-w_ representation of biological lipophilicity. Octanol–water system represents a simplified model of drug partitioning between aqueous and lipid phases, compared to the phospholipid bilayer of the brain cell membrane. P_oct-w_, for example, neglects the partitioning of charged molecules to phospholipids. A number of studies have demonstrated the correlation of P_oct-w_ and brain non-specific binding. P_oct-w_ was shown to explain about 52% (reported as R^2^) of the variability in experimentally-measured volume of distribution of unbound drug (V_u,brain_) in brain [14] and about 44–74% (reported as R^2^) of the variability in experimentally-measured fraction of unbound drug in brain (f_u,b_) [11–13]. This evidence indicates that P_oct-w_ provides an adequate predictor of brain non-specific binding.

### pH effect on drug ionization and its effect on drug transendothelial transport

Drug molecules in the CNS ionize depending on the compartment-specific pH and the drug-specific acid and base ionization constants. In LeiCNS-PK3.0, it is assumed that charged molecules can cross the barriers by paracellular diffusion only, ignoring the transcellular transport of charged species and paracellular route preference to cationic drugs [8]. Charged drug transcellular and paracellular transport rate is, however, negligible compared to neutral species transport rate and is not expected to critically influence LeiCNS-PK3.0 predictions.

### In vivo studies addressing the impact of CSF dynamics on brain ECF versus CSF PK profiles

A number of studies have supported the surrogacy of the CSF PK profiles to those of brain ECF, based on studies performed in rats, for both actively and passively transported drugs [2, 40, 41]. These studies are based, however, on CSF samples collected at the cisterna magna. Cranial CSF, including CSF at the cisterna magna, is in a relatively faster equilibrium with brain ECF, as compared to the distal lumbar CSF. In contrast to what is generally assumed, it has been shown in both in silico [42] and preclinical and clinical studies [43] that lumbar CSF does not reflect the PK profiles of brain ECF or even cisternal and ventricular CSF. In addition, our LeiCNS-PK3.0 sensitivity analysis suggests that brain ECF and ICF pharmacokinetic profiles are insensitive to CSF-related parameters. In a similar modeling study, the sensitivity analysis of a permeability-limited CNS PBPK model demonstrated that multiple factors while affecting the PK profiles of lumbar CSF, did not affect those of brain or even cranial CSF [42].

Preclinical and clinical studies that address the impact of altered CSF volume and/or flow on brain CSF PK profiles are rare, due to the associated technical and ethical restrictions. In addition, changing one CNS parameter in isolation is more of a hypothetical situation rather than can truly be realized in in vivo studies. Notwithstanding, a number of studies have addressed the impact of acetazolamide-induced reduction of CSF flow on brain ECF and CSF PK profiles. Acetazolamide is a carbonic anhydrase inhibitor drug, which reduces CSF production and flow by about 50%. Methotrexate exposure in the ventricular CSF of three patients was altered following acetazolamide administration, where the terminal elimination half-life increased [44] in agreement with the altered simulated profiles in Fig. 4 and supplementary Fig. 4 of this manuscript. The PK profile of alovudine measured in rat brain ECF with microdialysis was not altered in response to acetazolamide co-administration [45]. The PK profiles of 5-fluorouracil at rat brain ECF and cisterna magna CSF were altered to different extents following acetazolamide administration, implying the context dependency of drug concentration ratio of brain ECF to CSF [46]. It can be concluded as supported by LeiCNS-PK3.0 simulations and the in vivo preclinical and clinical studies that the lumbar CSF to brain ECF drug concentration ratio is context-dependent and this ratio might be altered in response to a change in CSF dynamics.

### Absence of CNS IIV and its implications

LeiCNS-PK3.0 accounts for interindividual variability (IIV) of the plasma pharmacokinetic parameters, but not that of the CNS physiology parameters. The impact of the IIV of CNS parameters on PK profiles is more prominent when drug transport is dependent on a certain parameter. For example, acetaminophen's, a slightly lipophilic and paracellularly-transported molecule, brain ECF PK profile is sensitive to the tight junction pore diameter (Supplementary Fig. 6). Thus, IIV of the tight junction pore diameter might account for the larger observed variability of brain ECF PK profile compared to that of plasma (Fig. 2, top panel). Acetaminophen PK profile while assuming nominal variabilities of 30% and 50% (as coefficient of variation, %CV) on physiological CNS parameters showed slightly wider 2.5th and 97.5th percentiles, which therefore better described the observed variability of the PK data (Supplementary Fig. 7).

The variability of the individual observed CNS concentrations relative to typical predicted profile was within three-fold error as indicated by %MARA. For humans, %MARA errors were 182%, 238% for brain ECF and SAS, respectively, while for rats these were 207%, 229%, and 216% for brain ECF, LV, and CM, respectively. Identification of variability of CNS model parameters and associated covariates is crucial for predicting the individual PK profiles, which remains challenging due to the limited data, e.g. on CNS physiology and measured drug concentrations, required for estimating this level of variability.

### Patho-pharmacokinetics require a systems approach

CNS drug exposure in healthy and diseased conditions is a function of both physiological and drug properties. In a healthy CNS, a number of mechanisms contribute to the rate and extent of the actual drug transport across the BBB, resulting in a brain ECF PK profile that may substantially differ from that of plasma. A change in any of the parameters that govern the PK at brain ECF and ICF, as identified by the sensitivity analysis, would potentially result in altered CNS drug exposure. This is particularly crucial in CNS diseases, in which the complex and multifactorial disease-specific pathophysiology would result in a distinct CNS PK profiles compared to those of a healthy CNS. In epilepsy, for instance, the increased expression of active efflux transporters at BBB is associated with a lower drug exposure in brain and hence resistance to therapy [47]. Furthermore, patients with traumatic brain injury showed higher morphine concentrations of the injured brain tissue ECF than those of the uninjured tissue, which is potentially due to decreased tight junction and active transporters expression at the BBB [7, 48]. Mechanistic, systems-based approaches such as PBPK modeling account for drug and CNS physiological properties in addition to the multidimensional disease pathology and are thus better suited for adequate PK predictions in healthy and diseased CNS. The shortage of knowledge on (patho-) physiological parameters and mechanisms remains a major challenge to translating CNS PBPK models between healthy and diseased populations.

### LeiCNS-PK3.0 applications

LeiCNS-PK3.0 applications include predicting PK profiles of small drugs in a healthy CNS and in patients with CNS diseases, e.g. Alzheimer’s, and exploring mechanistically the impact of CNS disease pathophysiology on CNS PK i.e. patho-pharmacokinetics. These applications are supported by mechanistic detailing of different physiological processes that for example distinguishes paracellular and transcellular transports, but also accounts for brain cells and lysosomes, a feature that was not supported in similar published CNS models [42, 49, 50]. LeiCNS-PK3.0 is thus useful at early stages of drug development to support (pre-) clinical study design and decision-making, e.g. dose selection and sampling time points.

## Conclusion

In conclusion, we improved our published LeiCNS-PK1.0 by accounting for brain non-specific binding and readdressing pH effect on drug ionization and passive transport. LeiCNS-PK3.0 simulations demonstrated that altered CSF dynamics changes brain ECF-to-SAS drug concentration ratio and implied a context-dependent PK surrogacy of lumbar SAS to brain ECF.

## Supplementary Information

Below is the link to the electronic supplementary material.

Supplementary file1 (DOCX 2611 kb)

## Data Availability

Data generated during and analyzed during the current study are not publicly available due privacy but are available from the corresponding author on reasonable request.
