# Supplementary material for: Lumbar cerebrospinal fluid-to-brain extracellular fluid surrogacy is context-specific: insights from LeiCNS-PK3.0 simulations
Source: J Pharmacokinet Pharmacodyn. 2021 Jun 17;48(5):725–41. doi: 10.1007/s10928-021-09768-7 (PMC8405486; doi:10.1007/s10928-021-09768-7)
Supplement: Supplementary file 1 — Supplementary file1 (DOCX 2611 kb) [file 10928_2021_9768_MOESM1_ESM.docx]

**Title: Lumbar cerebrospinal fluid-to-Brain extracellular fluid surrogacy is context-specific: insights from LeiCNS-PK3.0 simulations**

**Running title:** LeiCNS-PK3.0: a physiologically-based PK model to predict brain pharmacokinetics

**Journal:** Journal of Pharmacokinetics and Pharmacodynamics

Mohammed A. A. Saleh^1^, Chi Fong Loo^2^, Jeroen Elassaiss-Schaap^2,3^, Elizabeth C. M. de Lange^4^

1. Division of Systems Biomedicine and Pharmacology, Leiden Academic Center for Drug Research, Leiden University, Leiden, The Netherlands. ORCID ID: 0000-0002-0517-6051

2. Division of Systems Biomedicine and Pharmacology, Leiden Academic Center for Drug Research, Leiden University, Leiden, The Netherlands.

3. PD-value B.V., Houten, The Netherlands. ORCID ID: 0000-0002-3333-861X

4. Division of Systems Biomedicine and Pharmacology, Leiden Academic Center for Drug Research, Leiden University, Leiden, The Netherlands. ORCID ID: 0000-0001-8303-1117

Correspondence to email: [ecmdelange@lacdr.leidenuniv.nl](mailto:ecmdelange@lacdr.leidenuniv.nl), telephone: +31 71 527 6330

**Supplementary table 1:** Rat and human unbound drug concentrations sampled from different CNS locations and used to evaluate LeiCNS3 model.

|  | **Plasma** | **Brain_ECF_** | **CSF_LV_** | **CSF_CM_** | **CSF_SAS_** | **Total brain** | **Reference** |
| --- | --- | --- | --- | --- | --- | --- | --- |
| **Rat** | | | | | | | |
| Acetaminophen | X | X | X | X |  |  | [1] |
| Atenolol | X | X |  |  |  |  | [2] |
| Methotrexate | X | X | X | X |  |  | [3] |
| Morphine | X | X |  |  |  |  | [4, 5] |
| Paliperidone | X | X |  | X |  |  | [6] |
| Phenytoin | X | X |  |  |  |  | [6] |
| Quinidine | X | X | X | X |  | X | [7] |
| Raclopride | X | X |  |  |  | X | [8] |
| Remoxipride | X | X | X | X |  | X | [6, 9, 10] |
| Risperidone | X | X |  | X |  |  | [6] |
| **Human** | | | | | | | |
| Acetaminophen | X |  |  |  | X |  | [11, 12] |
| Indomethacin | X |  |  |  | X |  | [13, 14] |
| Morphine | X | X |  |  |  |  | [15] |
| Oxycodone | X |  |  |  | X |  | [16] |

**Supplementary table 2:** LeiCNS-PK3.0 physiological parameters values of rats and humans

| **Species** | | **Rat values** | | **Human values** | |
| --- | --- | --- | --- | --- | --- |
| **Parameter** | | **Value**  **(range)** | **Reference** | **Value**  **(range)** | **Reference** |
| **Volumes (mL)** | Total brain (V_tot_) | 1.8^[[1]](#footnote-1)^ | [17, 18] | 1250  (1110 - 1380) | [19–22] |
|  | Brain extracellular fluid (V_ECF_) | 0.36^[[2]](#footnote-2)^ | [23] | 253  (217 - 300)^b^ | [23–27] |
|  | Brain intracellular fluid (V_ICF_) | 1.44^[[3]](#footnote-3)^ | [23] | 1000^c^ |  |
|  | Brain cell lysosomes (V_LYS_) | 0.018^[[4]](#footnote-4)^ | [28] | 12.5^d^ | [28] |
|  | Brain microvasculature (V_MV_) | 0.054^[[5]](#footnote-5)^ | [29] | 45  (37 - 50)^[[6]](#footnote-6)^ | [25, 30, 31] |
|  | Total cerebrospinal fluid (V_CSF_) | 0.28^[[7]](#footnote-7)^  (0.155 – 0.4) | [32–34] | 140 |  |
|  | Lateral ventricles (V_LV_) | 0.0075^[[8]](#footnote-8)^  (0.003 – 0.015) | [33, 35–37] | 20  (11 – 16) | [38–42] |
|  | 3^rd^ & 4^th^ ventricles (V_TFV_) | 0.0075  (0.003 – 0.015) |  | 3  (2.3 – 3.7) | [40, 41] |
|  | Cisterna magna (V_CM_) | 0.017^[[9]](#footnote-9)^ | [3, 43] | 1 | [44] |
|  | Subarachnoid space (V_SAS_) | 0.135^[[10]](#footnote-10)^ | [45] | 116  (110-116) | [46–48] |
| **Flows (mL min^-1^)** | Cerebral blood flow (Q_CBF_) | 2.87^[[11]](#footnote-11)^ | [29, 49] | 689  (644-722) | [50–52] |
|  | Brain ECF bulk flow (Q_ECF_) | 0.0002  (0.18E^-3^ - 0. 2E^-3^) | [53–55] | 0.2^[[12]](#footnote-12)^ | [56] |
|  | CSF flow (Q_CSF_) | 0.0022  (0.18E^-2^ - 0.22E^-2^) | [33, 57] | 0.42  (0.28-0.68) | [48, 58–61] |
| **Surface areas (cm^2^)** | Blood brain barrier (SA_BBB_) | 155  (150 - 188) | [62–64] | 150000  (140 E^3^-360 E^3^) | [65–73] |
|  | Blood CSF barrier (SA_BCSFB_) | 25^[[13]](#footnote-13)^ | [62] | 15000^[[14]](#footnote-14)^ | [74, 75] |
|  | Brain cell membrane (SA_BCM_) | 4250^[[15]](#footnote-15)^ | [76] | 2666520^o^ | [77, 78] |
|  | Lysosomes membrane (SA_LYS_) | 2700^[[16]](#footnote-16)^ | [79] | 1980260^[[17]](#footnote-17)^ | [79–83] |
| **Width**  **(µm)** | Blood brain barrier (W_BBB_) | 0.5  (0.2-0.5) | [84] | 0.5 (0.2-0.4) | [70, 85] |
|  | Blood CSF barrier (W_BCSFB_) |  |  |  |  |
| **Number** | Total brain cells (N_br,cells_) | 3.32E^8^ | [76] | 1.71E^11^ ^[[18]](#footnote-18)^ | [77, 78] |
| **Pore size**  **(µm)** | Blood brain barrier (pTJ_BBB_) | 0.001 | [86] | 0.0007  (0.0008- 0.001) | [86, 87] |
|  | Blood CSF barrier (pTJ_BCSFB_) | 0.009 | [86] | 0.0027 | [86] |
| **Effective surface area  (%)** | BBB Transcellular transport (SA_BBB,T_) | 99.8^[[19]](#footnote-19)^ | [9, 88, 89] | 99.8^[[20]](#footnote-20)^ | [9, 88, 89] |
|  | BCSFB Transcellular transport (SA_BCSFB,T_) | 99.8s |  | 99.8t |  |
|  | BBB paracellular transport (SA_BBB,P_) | 0.006^[[21]](#footnote-21)^ | [86] | 0.004u | [86] |
|  | BCSFB paracellular transport (SA_BCSFB,P_) | 0.05^u^ | [86] | 0.016u |  |
| **pH** | Plasma (pH_PL_) | 7.4 | [90] | 7.4 | [90] |
|  | Brain microvasculature (pH_MV_) |  |  |  |  |
|  | Brain extracellular fluid (pH_ECF_) | 7.3 | [91] | 7.3 | [91] |
|  | Cerebrospinal fluid (pH_CSF_) | 7.3 | [92] | 7.3 | [92] |
|  | Brain cells (pH_ICF_) | 7 | [91] | 7 | [91] |
|  | Brain cell lysosomes (pH_LYS_) | 5 | [91] | 5 | [91] |

**Supplementary equations**

Paracellular clearance across the blood-brain (BBB) and blood-cerebrospinal fluid (BCSFB) barriers (Qp_BBB/BCSFB_) was calculated using aqueous diffusivity of the drug (Daq, cm^2^/sec), the molecular weight of the drug (MW, in g/mol), diffusion width across BBB/BCSFB (width_BBB/BCSFB_), and surface areas (SAp_BBB/BCSFB_ ) of BBB and BCSFB. BBB and BCSFB surface areas were corrected with effective surface area factors for BBB and BCSFB (Supplementary table 2) to account for surface area dedicated for paracellular transport.

$$Daq= -4.113-0.4609*logMW$$

$${Qp}_{BBB/BCSFB}=\frac{Daq}{{width}_{BBB/BCSFB}}*{SAp}_{BBB/BCSFB}$$

Transcellular clearance across the blood-brain (BBB) and blood-cerebrospinal fluid (BCSFB) barriers (Qt_BBB/BCSFB_) was calculated based on transmembrane permeability of the drug (P_0_^transcellular^, cm/sec), octanol-water partition coefficient (logP), and surface areas (SAt_BBB/BCSFB_ ) of BBB and BCSFB. BBB and BCSFB surface areas were corrected with effective surface area factors for BBB and BCSFB (Supplementary table 2) to account for surface area dedicated for transcellular transport only.${logP}_{0}^{transcellular}= 0.939*logP-6.21$

$${Qt}_{BBB/BCSFB}= 0.5* P_{0}^{transcellular}*{SAt}_{BBB/BCSFB}$$

Equation calculating the influx and efflux asymmetry factors at the BBB and BCSFB:

$${AF}_{BBB, in}=-\frac{{Kp}_{uu, ECF}*Q_{CBF}*Q_{ECF}+{{Kp}_{uu, CM}*Qp}_{BBB}*Q_{CSF}+Q_{CBF}*({Kp}_{uu, ECF}*({AF}_{BBB, ef}*{PHF}_{ECF}*{Qt}_{BBB}+{Qp}_{BBB})-{Qp}_{BBB})}{{PHF}_{MV}*{Qt}_{BBB}*({Kp}_{uu,CM}*Q_{CSF}-Q_{CBF})}$$

$${AF}_{BBB, ef}=-\frac{{Kp}_{uu, ECF}*Q_{CBF}*Q_{ECF}+{Kp}_{uu, CM}*Q_{CSF}*({AF}_{BBB, in}*{PHF}_{MV}*{Qt}_{BBB}+{Qp}_{BBB})+Q_{CBF}*({Kp}_{uu, ECF}*{Qp}_{BBB}-{AF}_{BBB, in}*{PHF}_{MV}*{Qt}_{BBB}-{Qp}_{BBB})}{{Kp}_{uu, ECF}*{PHF}_{ECF}*{Qt}_{BBB}*Q_{CBF}}$$

$${AF}_{LV, in} =\frac{{Kp}_{uu, ECF}*Q_{CBF}*Q_{ECF}-Q_{CSF}*\left( {Kp}_{uu, LV}*Q_{CBF}+{Kp}_{uu, CM}*{Qp}_{BCSFB} \right)-Q_{CBF}*({Kp}_{uu, LV}*\left( {AF}_{LV, ef}*{PHF}_{LV}*{Qt}_{BCSFB}+{Qp}_{BCSFB} \right)-{Qp}_{BCSFB})}{{PHF}_{MV}*{Qt}_{BCSFB}*({Kp}_{uu, CM}*Q_{CSF}-Q_{CBF})}$$

$${AF}_{LV, ef} =\frac{{Kp}_{uu, ECF}*Q_{CBF}*Q_{ECF}-Q_{CSF}*\left( {Kp}_{uu, CM}*\left( {AF}_{LV, in}*{PHF}_{MV}*{Qt}_{BCSFB}+{Qp}_{BCSFB} \right)+{Kp}_{uu, LV}*Q_{CBF} \right)+Q_{CBF}*(-{Kp}_{uu, LV}*{Qp}_{BCSFB}+{AF}_{LV, in}*{PHF}_{MV}*{Qt}_{BCSFB}+{Qp}_{BCSFB})}{{Kp}_{uu, LV}*{PHF}_{LV}*{Qt}_{BCSFB}*Q_{CBF}}$$

$${AF}_{TFV, in} =\frac{Q_{CSF}*\left( Q_{CBF}*\left( {Kp}_{uu, LV}-{Kp}_{uu, CM} \right)-{Kp}_{uu, CM}*{Qp}_{BCSFB} \right)+Q_{CBF}*({-Kp}_{uu, CM}*({AF}_{TFV, ef}*{PHF}_{TFV}*{Qt}_{BCSFB}+{Qp}_{BCSFB})+{Qp}_{BCSFB})}{{PHF}_{MV}*{Qt}_{BCSFB}*({Kp}_{uu, CM}*Q_{CSF}-Q_{CBF})}$$

$${AF}_{TFV, ef} =\frac{Q_{CSF}*\left( Q_{CBF}*\left( {Kp}_{uu, LV}-{Kp}_{uu, CM} \right)-{Kp}_{uu, CM}*\left( {AF}_{TFV, in}*{PHF}_{MV}*{Qt}_{BCSFB}+{Qp}_{BCSFB} \right) \right)+Q_{CBF}*(-{Kp}_{uu, CM}*{Qp}_{BCSFB}+{AF}_{TFV, in}*{PHF}_{MV}*{Qt}_{BCSFB}+{Qp}_{BCSFB})}{{Kp}_{uu, CM}*{PHF}_{TFV}*{Qt}_{BCSFB}*Q_{CBF}}$$

Supplementary equations notations

AF_BBB,ef_: efflux asymmetry factor across the blood brain barrier

AF_BBB,in_: influx asymmetry factor across the blood brain barrier

AF_LV,ef_: efflux asymmetry factor across the blood cerebrospinal fluid barrier at lateral ventricles

AF_LV,in_: influx asymmetry factor across the blood cerebrospinal fluid barrier at lateral ventricles

AF_TFV,ef_: efflux asymmetry factor across the blood cerebrospinal fluid barrier at 3^rd^ and 4^th^ ventricles

AF_TFV,in_: influx asymmetry factor across the blood cerebrospinal fluid barrier at 3^rd^ and 4^th^ ventricles

Kp_uu,CM_: cisterna magna-to-plasma unbound drug concentration ratio

Kp_uu,ECF_: brain extracellular fluid-to-plasma unbound drug concentration ratio

Kp_uu,LV_: lateral ventricles-to-plasma unbound drug concentration ratio

PHF_ECF_: pH factor of brain extracellular fluid

PHF_LV_: pH factor of lateral ventricles

PHF_MV_: pH factor of brain microvasculature

PHF_TFV_: pH factor at 3^rd^ and 4^th^ ventricles

Q_CBF_: cerebral blood flow

Q_CSF_: cerebrospinal fluid flow

Q_ECF_: brain extracellular fluid bulk flow

Qp_BBB_: paracellular transport clearance at blood brain barrier

Qp_BCSFB_: paracellular transport clearance at blood cerebrospinal fluid barrier

Qt_BBB_: transcellular transport clearance at blood brain barrier

Qt_BCSFB_: transcellular transport clearance at blood cerebrospinal fluid barrier

**Supplementary fig. 1** Detailed mathematical structure of LeiCNS-PK3.0. LeiCNS-PK3.0 is composed of whole body empirical plasma model and CNS PBPK model. Both models communicate via cerebral blood flow.


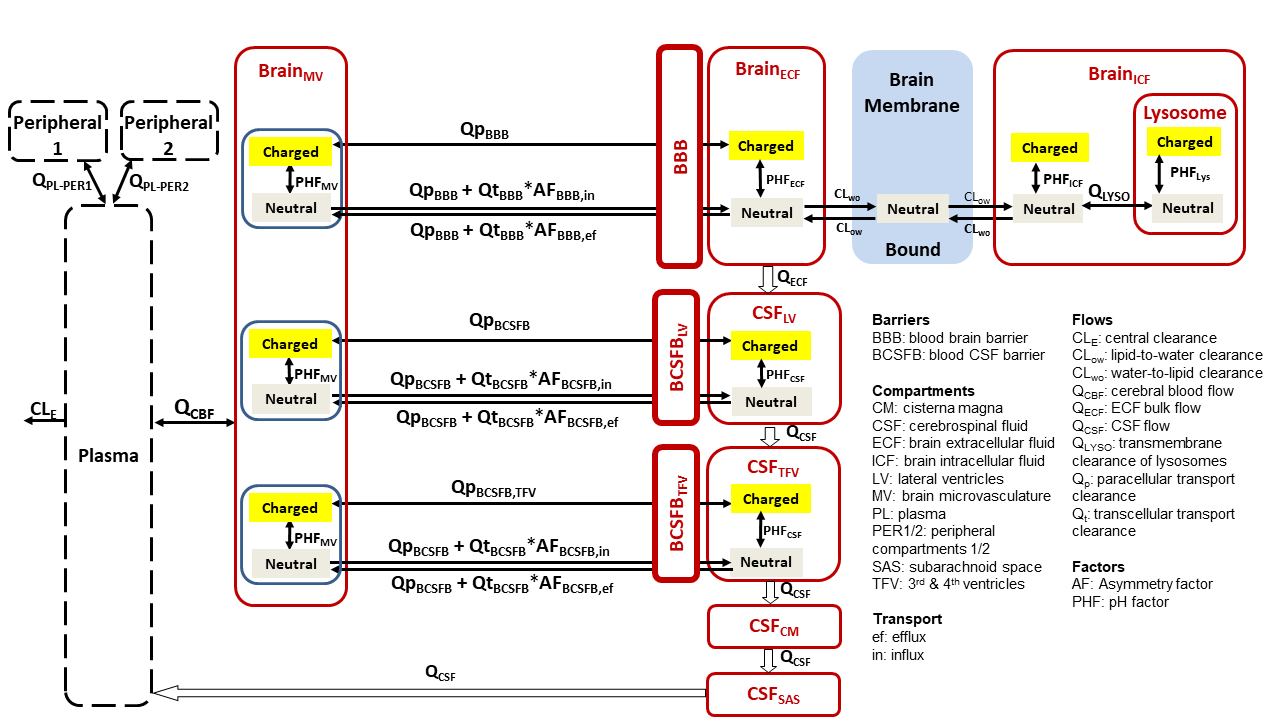


**Supplementary fig. 2** Model evaluation of the rat LeiCNS-PK3.0 model. A-B) Visual predictive checks plots compared in vivo measured drug concentration (black dots) in multiple CNS locations to the median (solid line) and 95% prediction intervals (colored band) of 200 model simulations. C) Boxplot of the relative accuracy error calculated for different drugs. Green and yellow solid lines represent two- and five- fold error, respectively. ECF: brain extracellular fluid, LV: lateral ventricles, CM: cisterna magna, BH: brain homogenate.

**(a)**

**
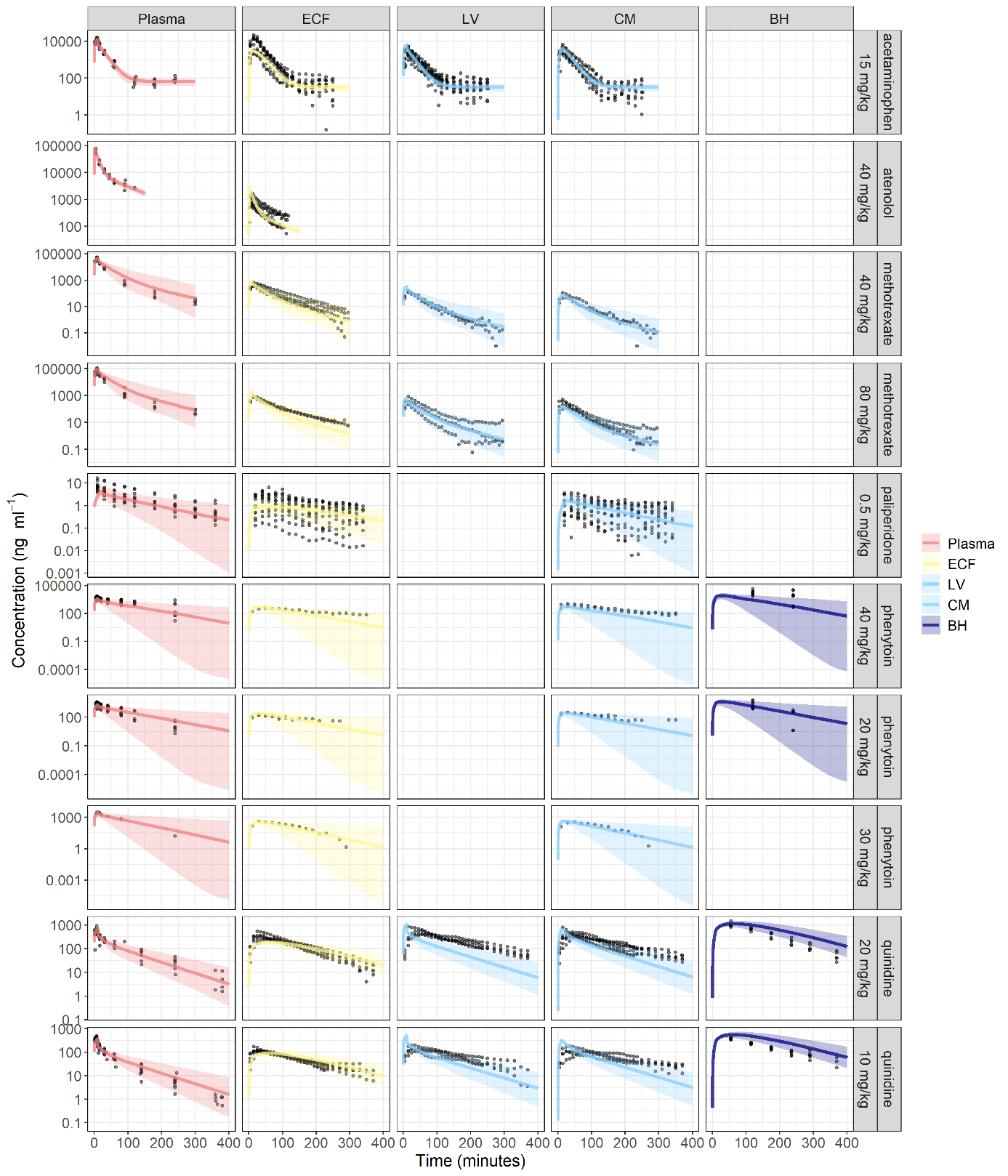
**

**(b)**

**
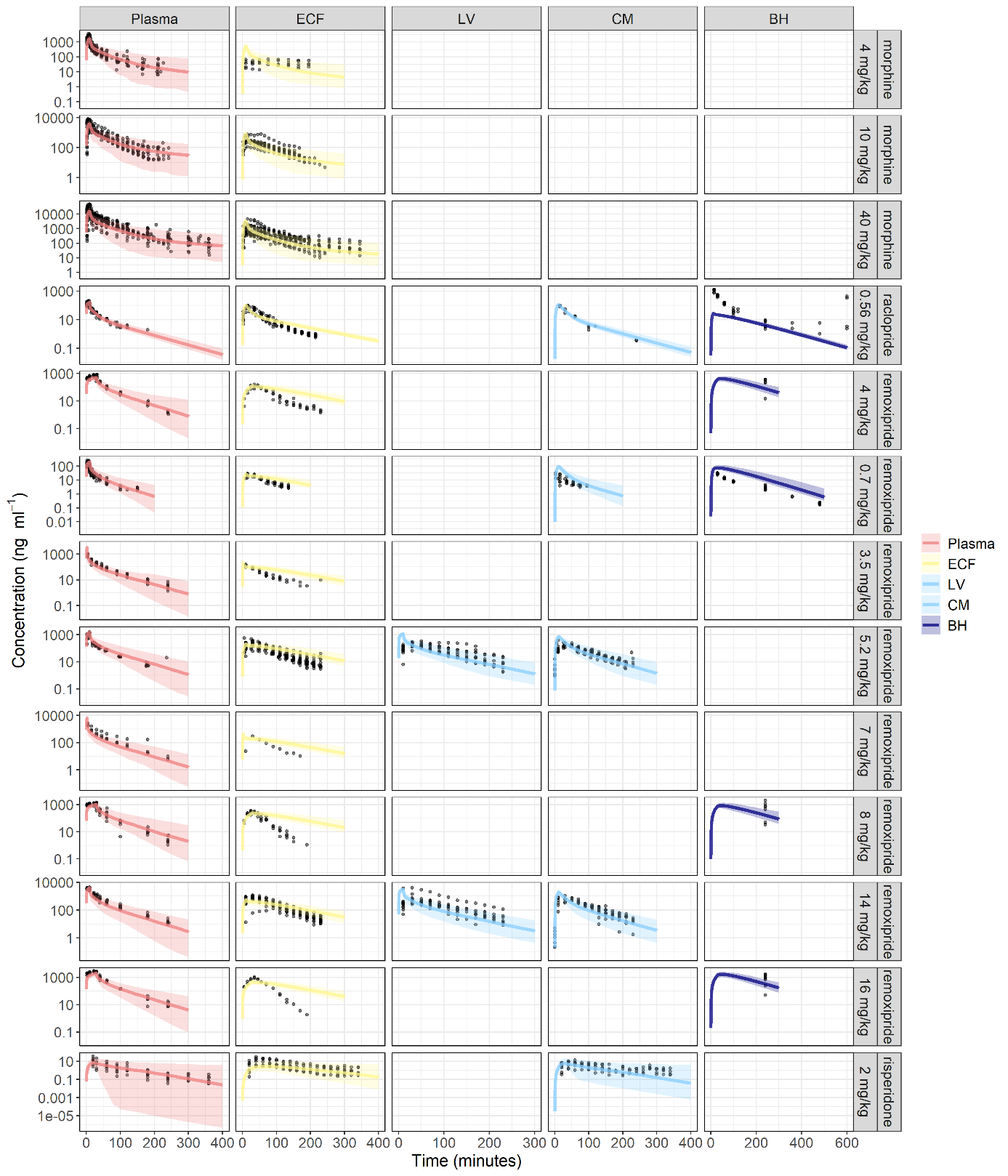
**


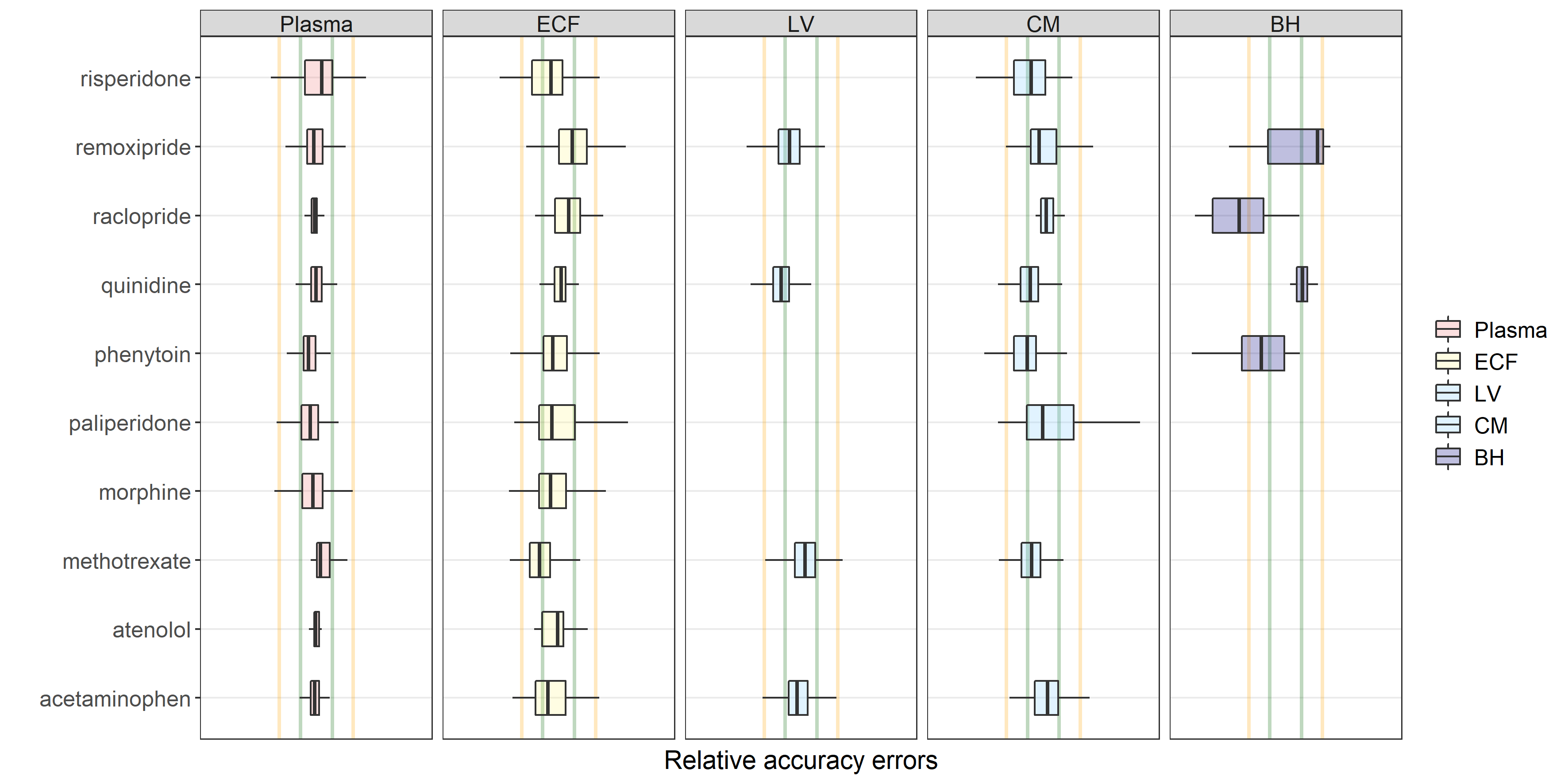
**(c)**

**Supplementary fig. 3:** Model evaluation of the human LeiCNS-PK3.0 model. Boxplot of the relative accuracy error calculated for different drugs. Green and yellow solid lines represent two- and five- fold error, respectively. ECF: brain extracellular fluid, SAS: subarachnoid space.


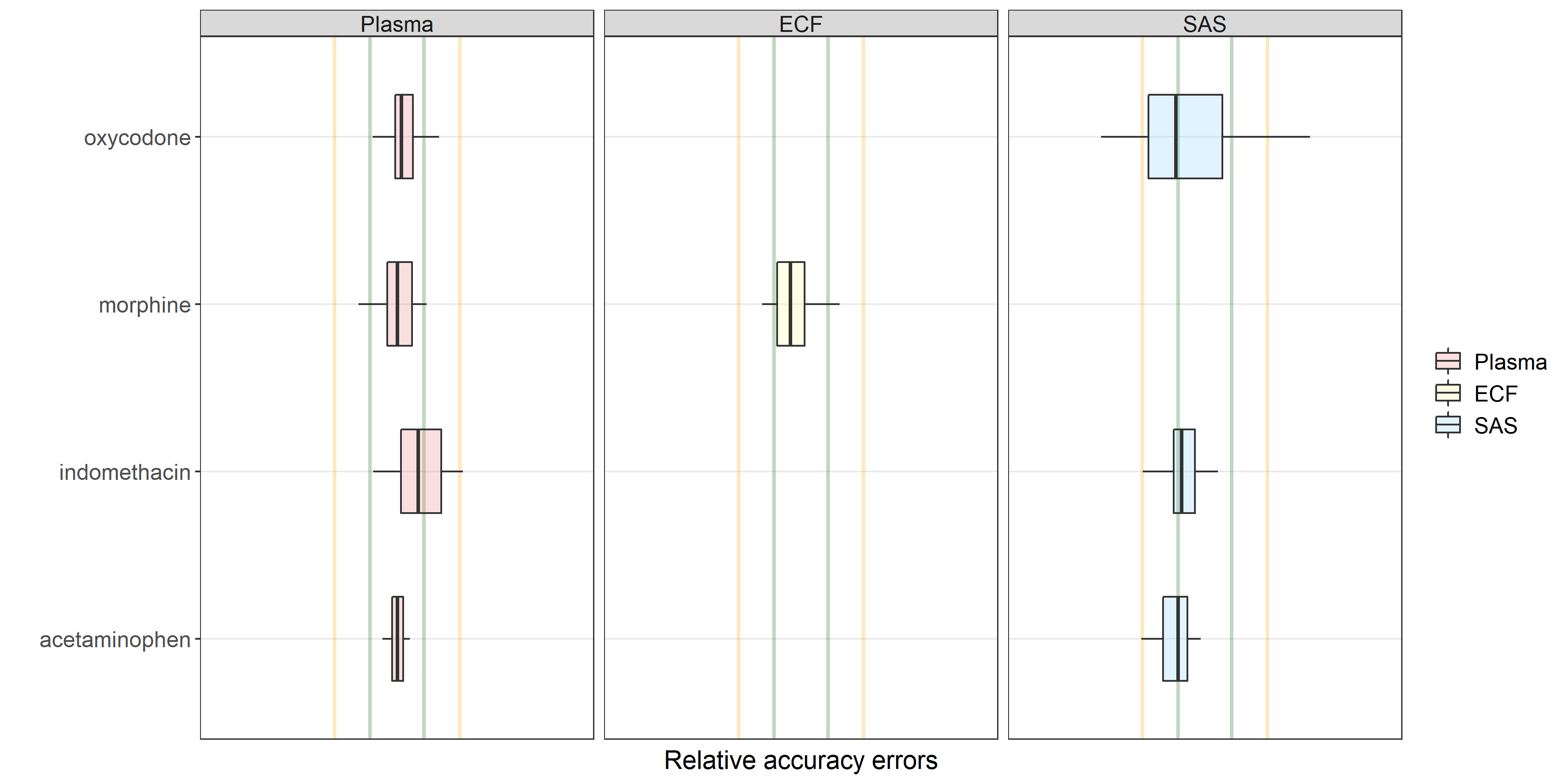


**Supplementary fig. 4 a-e** Pharmacokinetic profiles of test drugs at brain extracellular (ECF) fluid and subarachnoid space (SAS) at physiologic and two-fold altered cerebrospinal fluid (CSF) volume and flow. Changing CSF dynamics affects SAS pharmacokinetics and not brain ECF pharmacokinetics. Test drugs included methotrexate, phenytoin, atenolol, raclopride, and risperidone. ECF: brain extracellular fluid, SAS: subarachnoid space.

a)


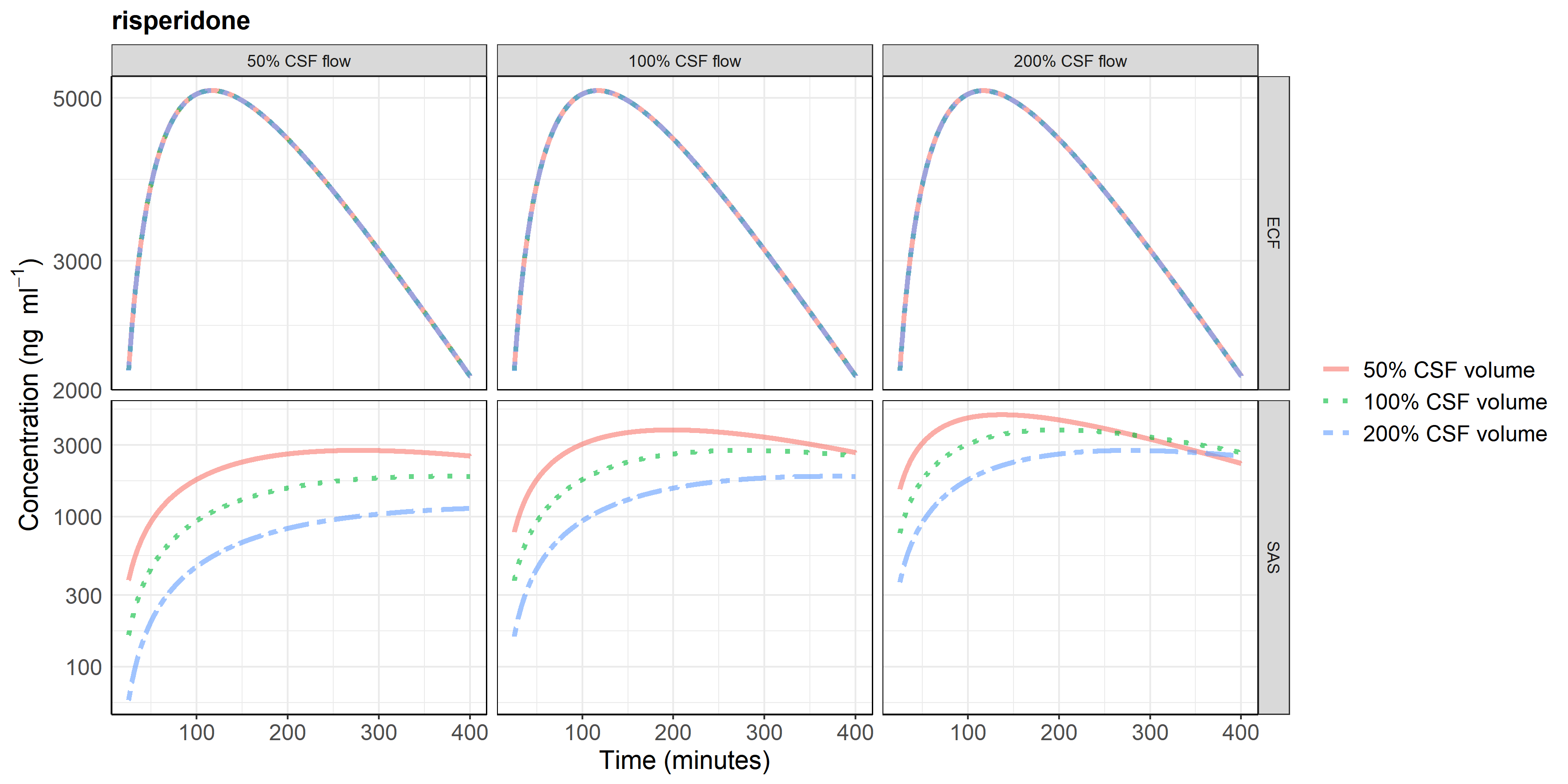


b)
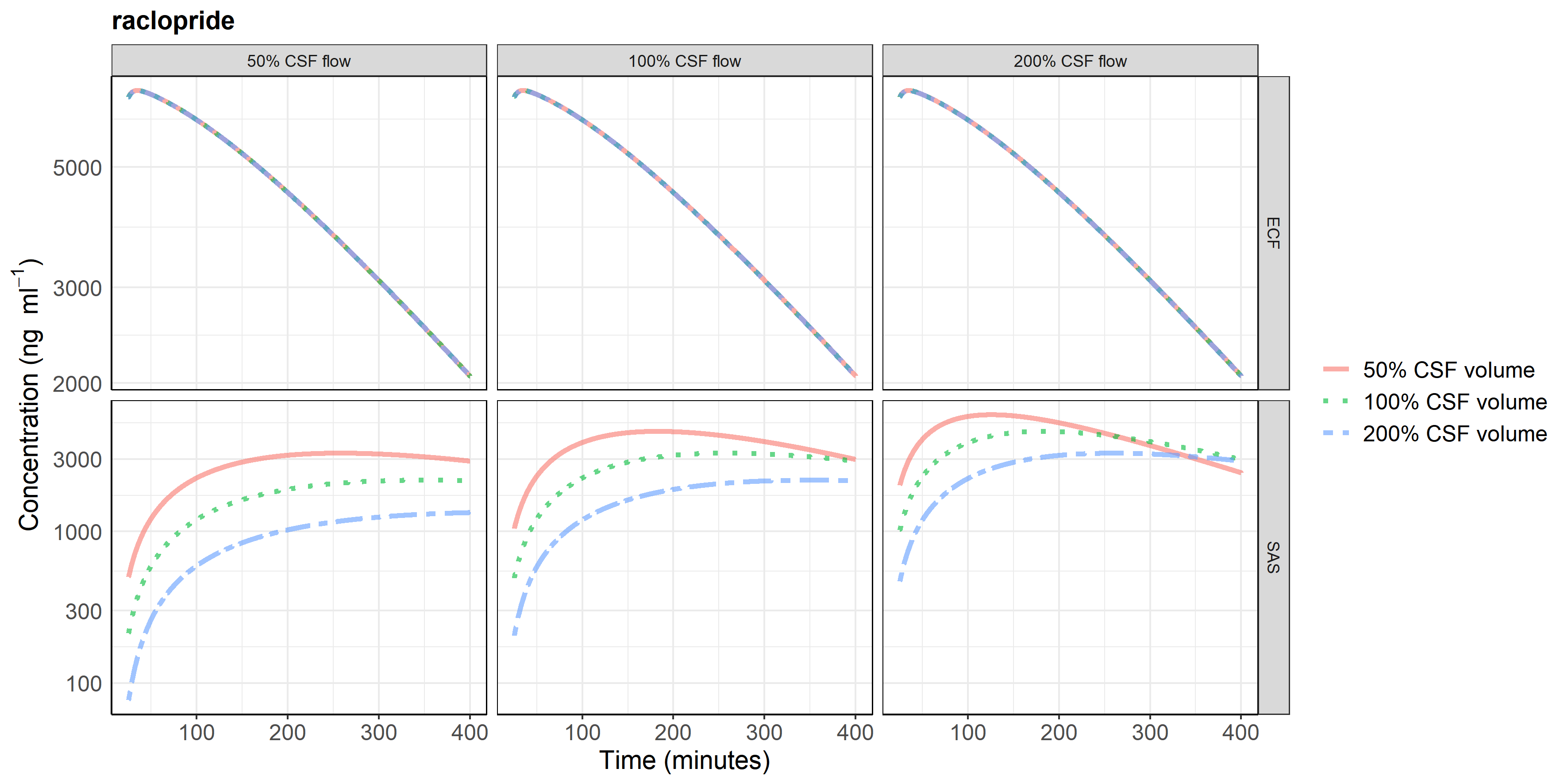


c)
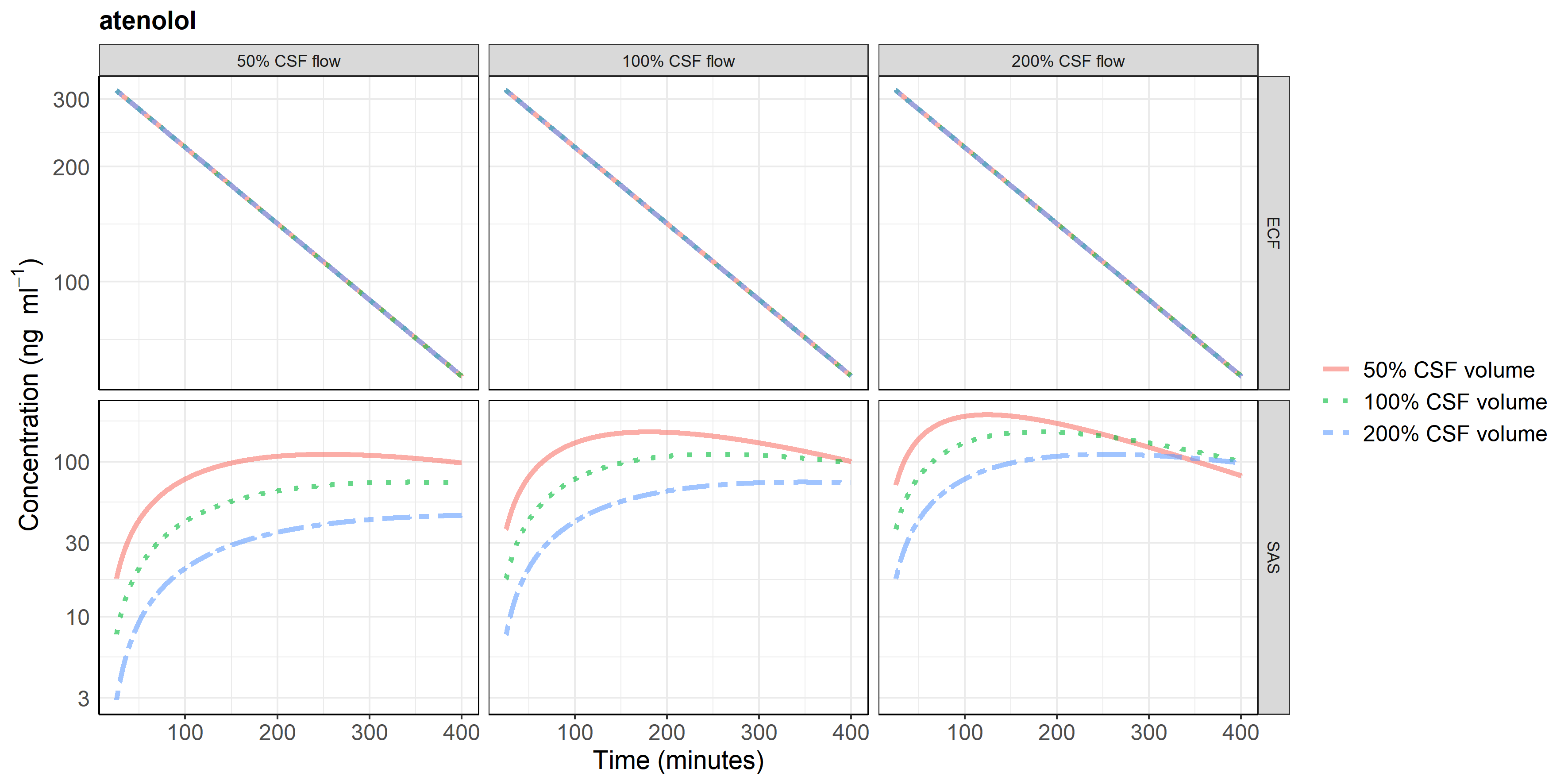


d)
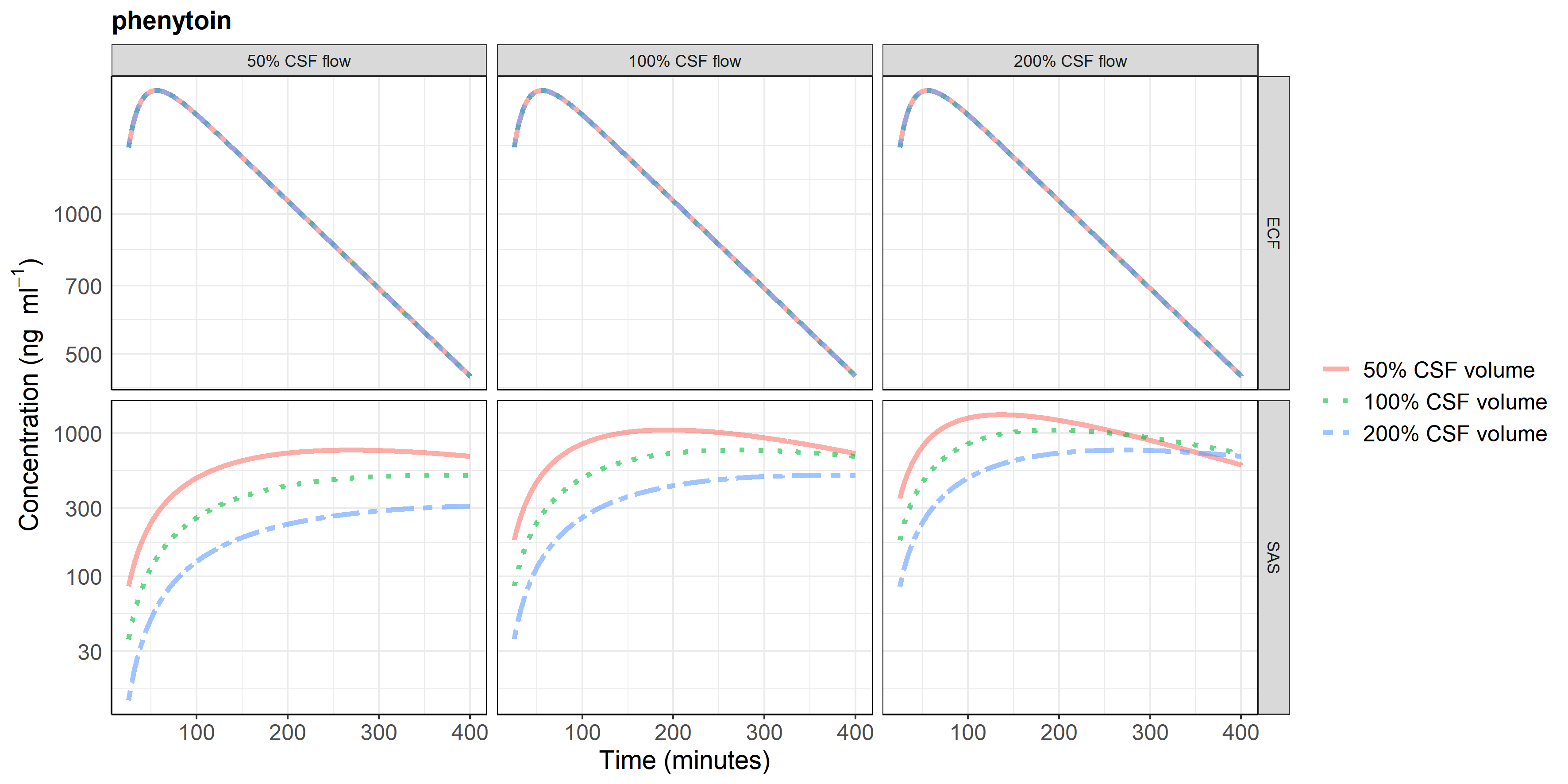


e)
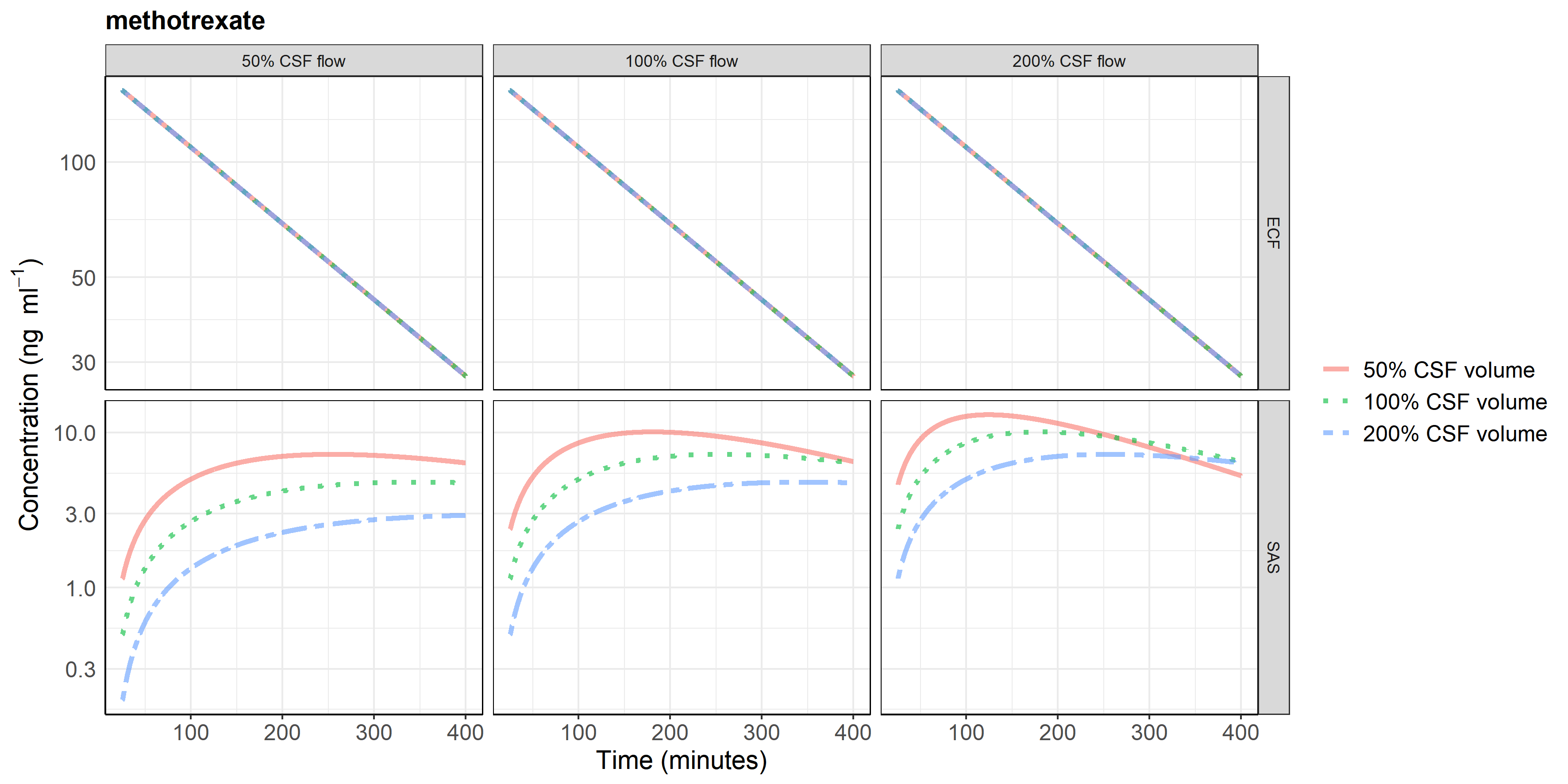


**Supplementary fig. 5 a-e** Pharmacokinetic profiles of test drugs at brain extracellular (ECF) fluid and subarachnoid space (SAS) at physiologic and five-fold altered cerebrospinal fluid (CSF) volume and flow. Changing CSF dynamics affects SAS pharmacokinetics and not brain ECF pharmacokinetics. Test drugs included methotrexate, phenytoin, atenolol, raclopride, and risperidone. ECF: brain extracellular fluid, SAS: subarachnoid space.

a)
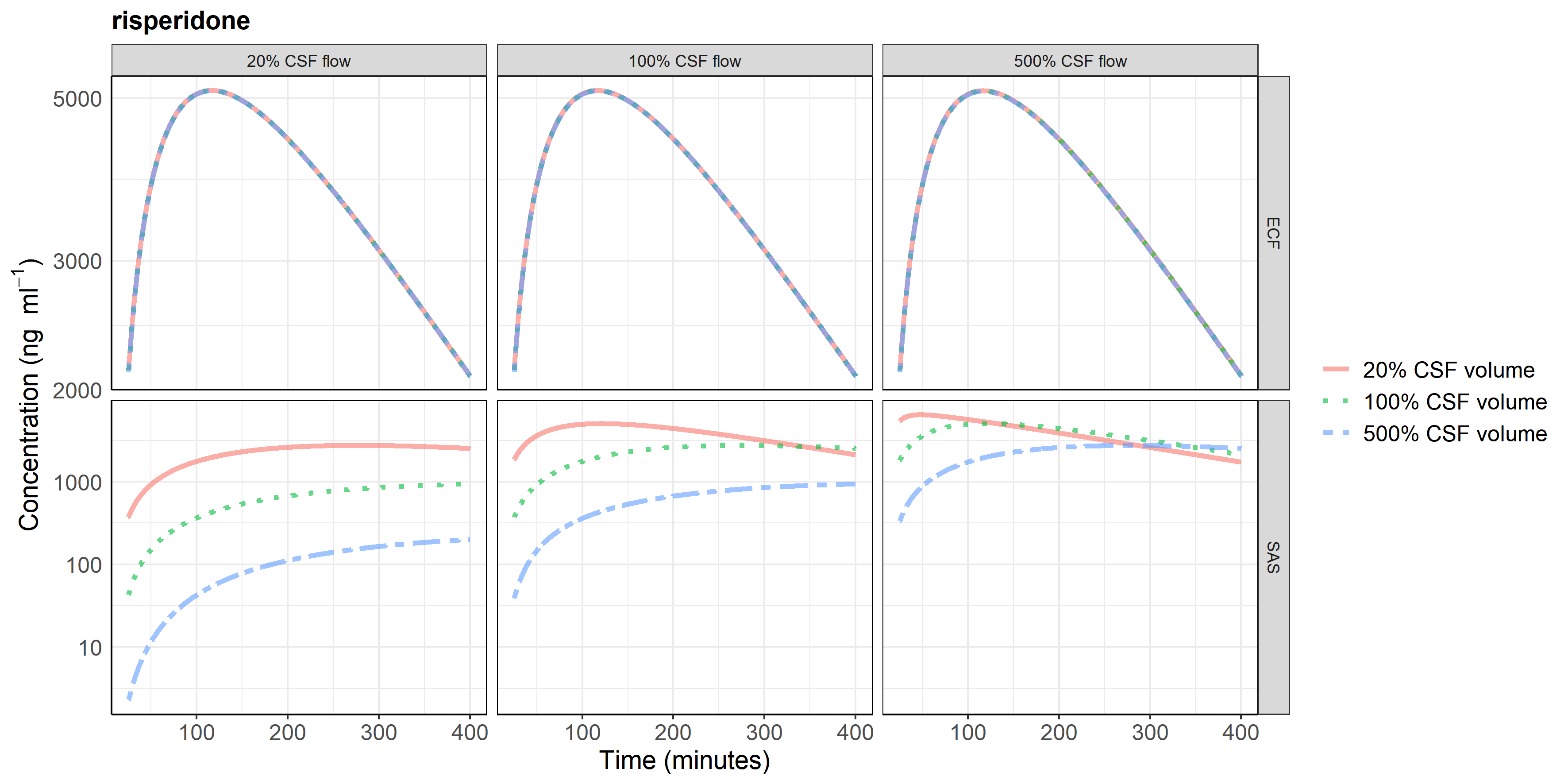


b)
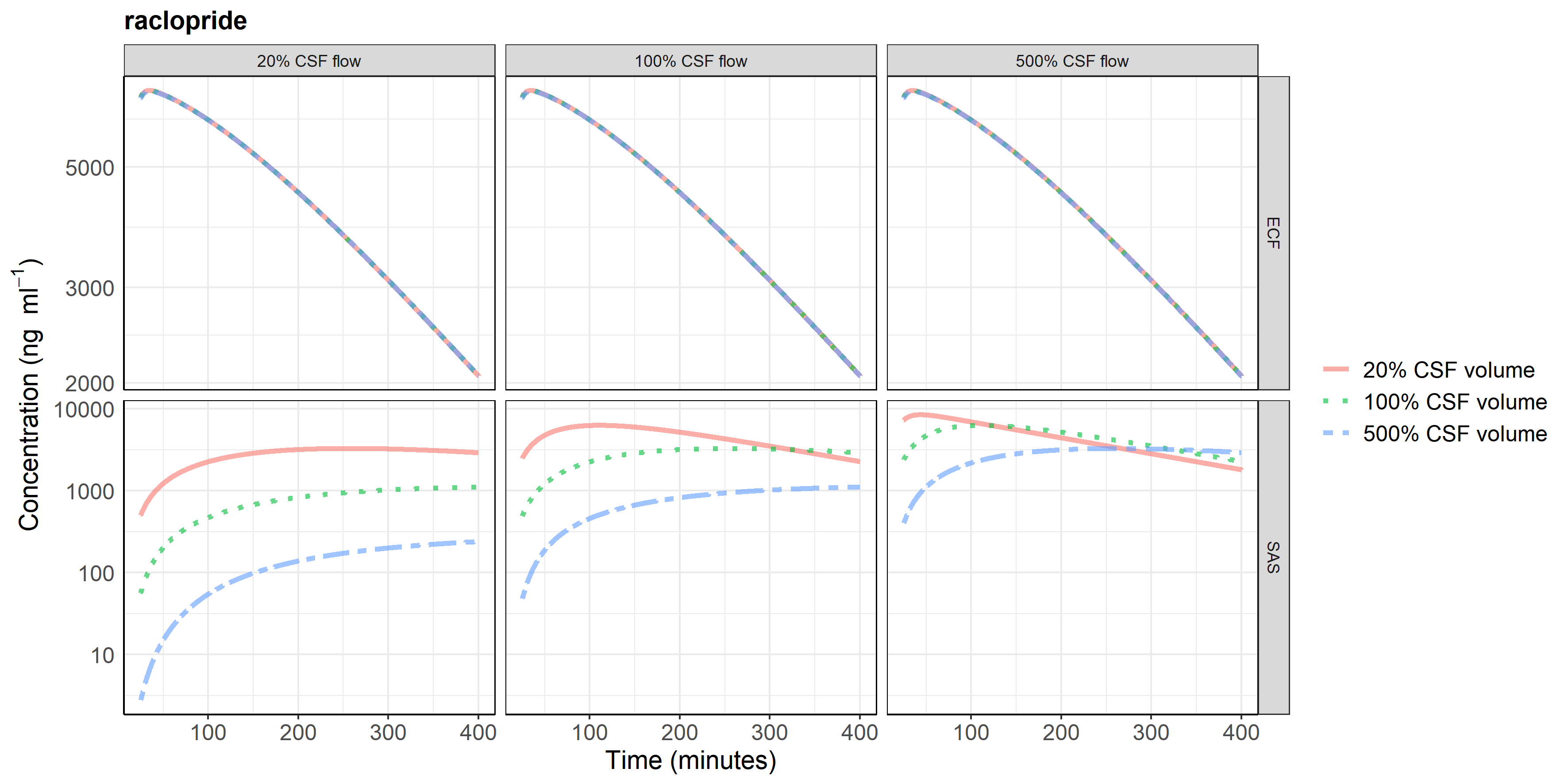


c)
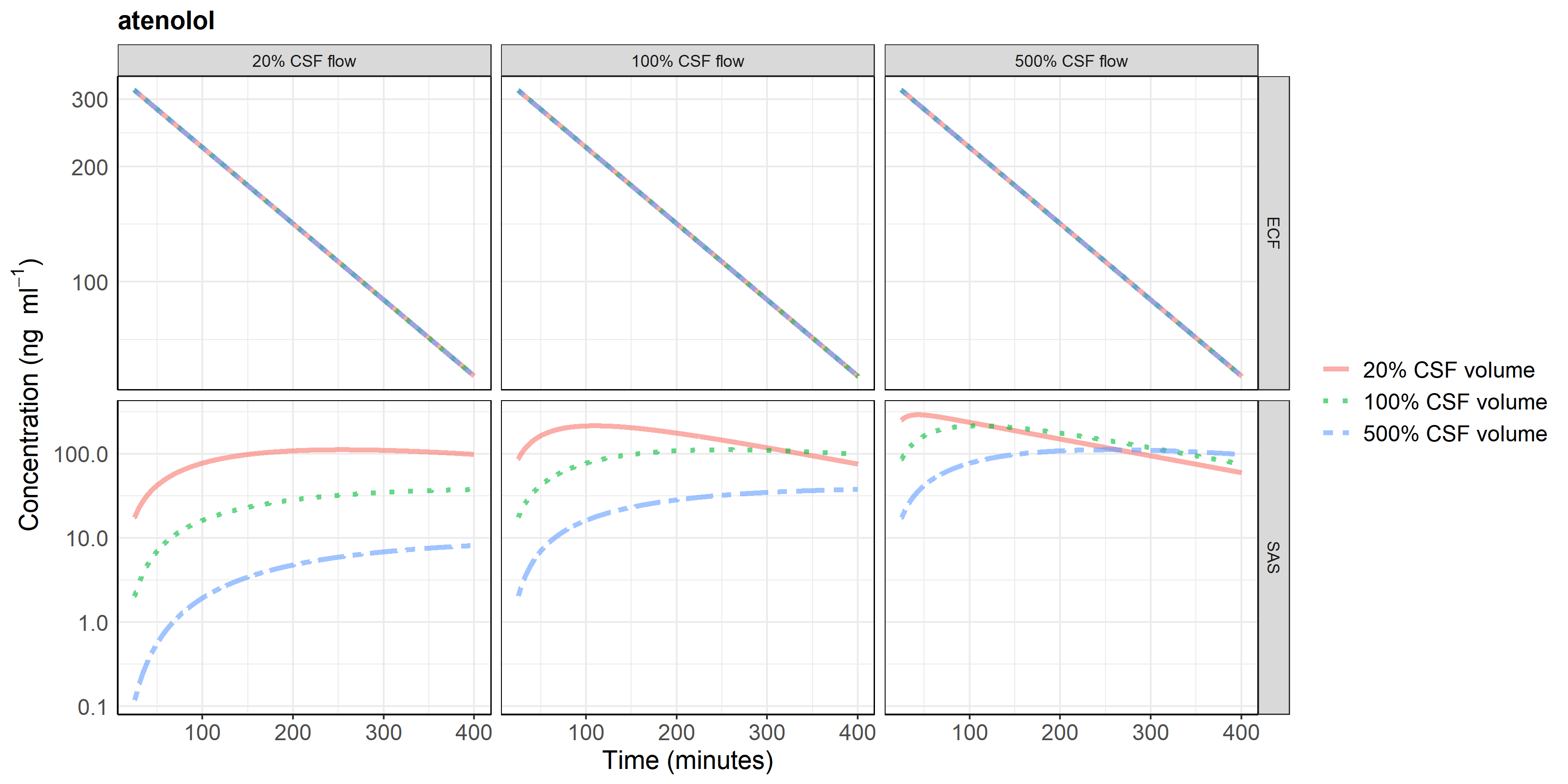


d)
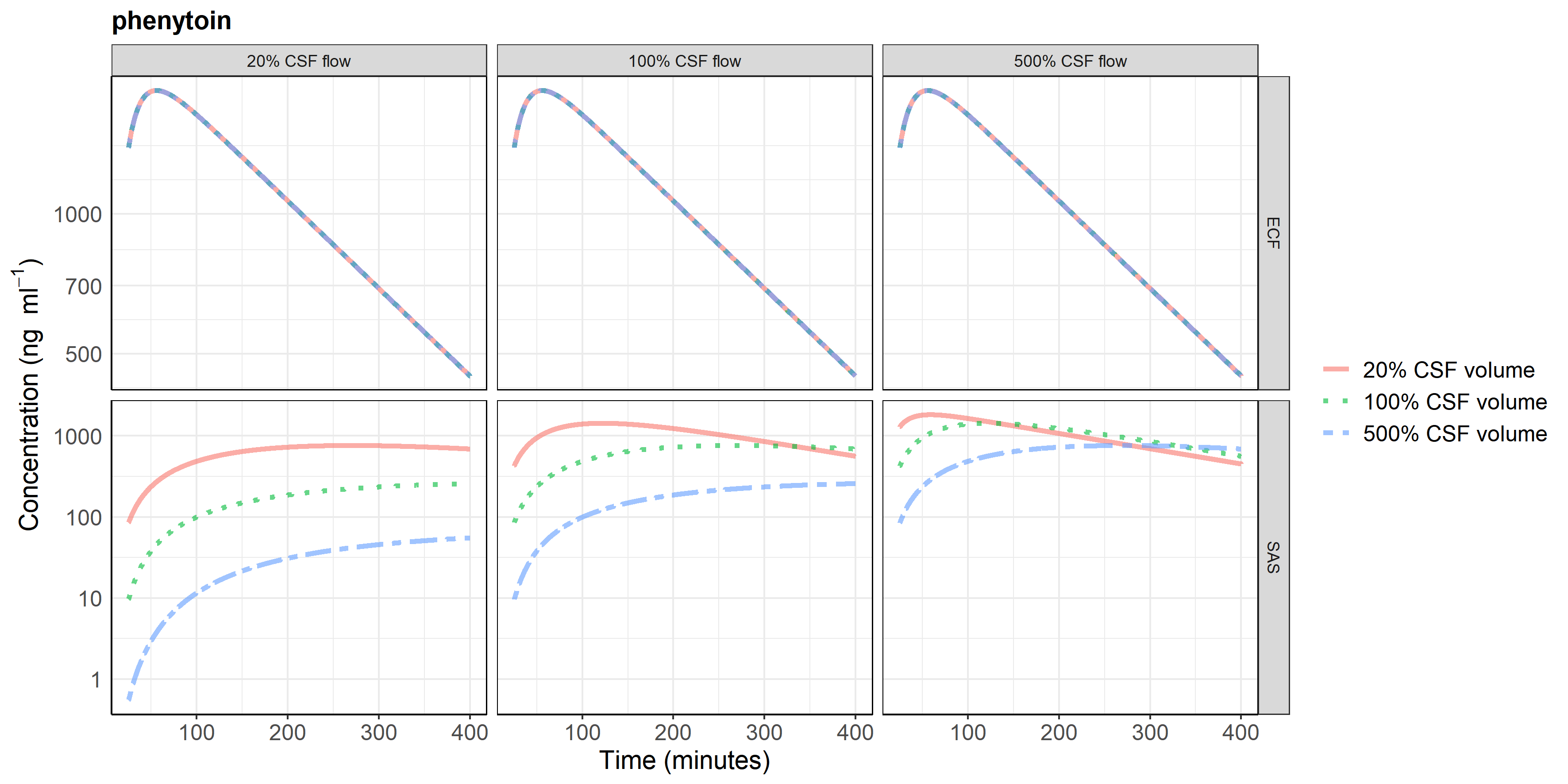


e)
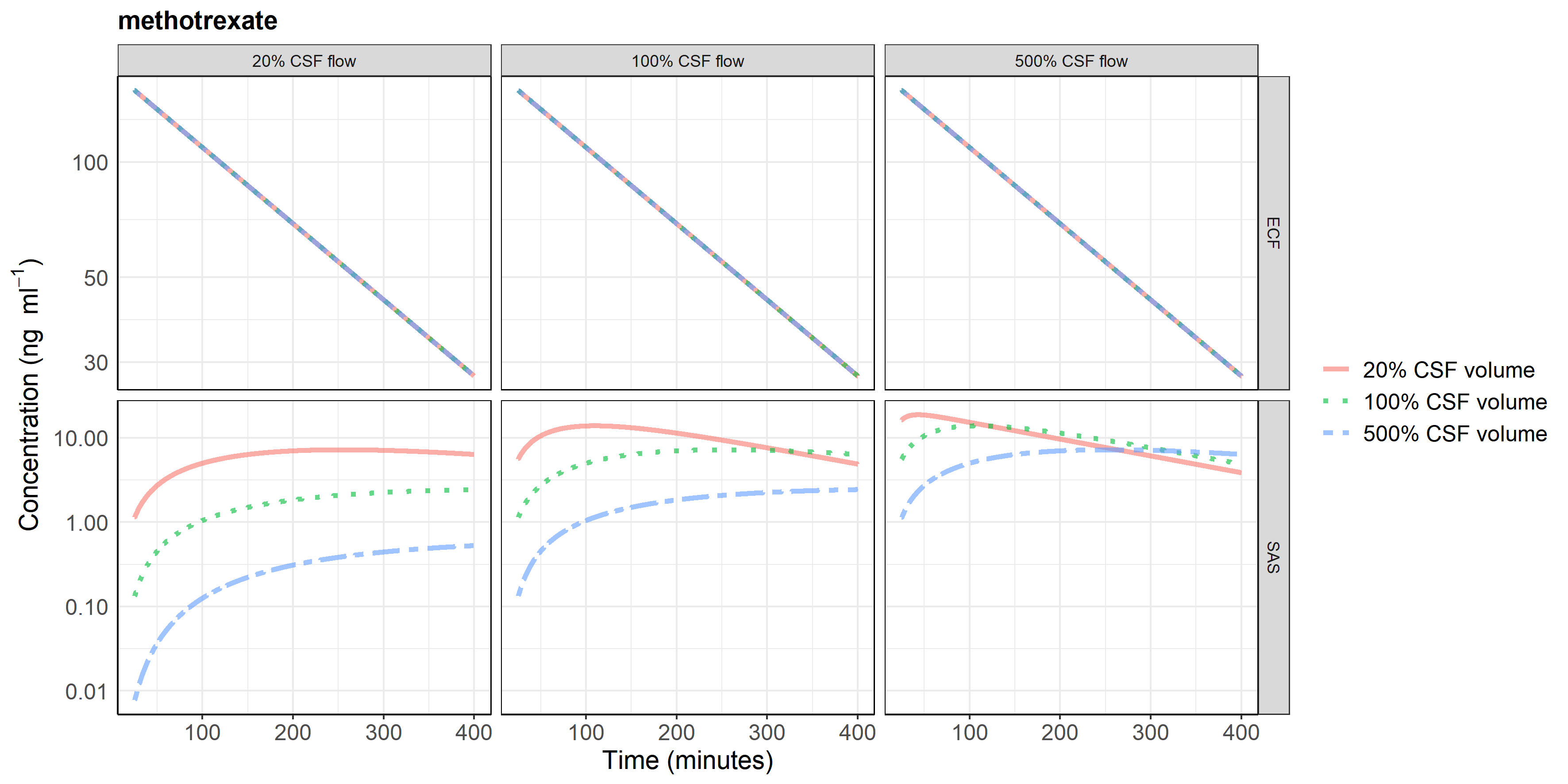


**Supplementary fig. 6** LeiCNS-PK3.0 sensitivity analysis results. The heatmap highlights that CNS parameters that impact the drug pharmacokinetic profile at the brain extracellular fluid, brain cells, and subarachnoid space. The colored scale is log_2_ transformed and thus 1 indicate two-fold higher outcome. AUC: area under the concentration-time curve, C_max_: maximum concentration, kp_uu,ECF_: brain ECF to plasma unbound drug ratio, kp_uu,CM_: cisterna magna to plasma unbound drug ratio, T_max_: time at which C_max_ is achieved, PHF_ECF_: pH factor of brain extracellular fluid, PHF_ICF_: pH factor of brain cells, Q_CBF_: cerebral blood flow, Q_CSF_: cerebrospinal fluid flow, SA_BBB,p_: effective surface area of paracellular transport route, SA_BCM_: surface area of brain cell membrane, V_ECF_: volume of brain extracellular fluid, V_ICF_: volume of brain cells, V_PhB_: volume fraction of brain phospholipid, V_SAS_: subarachnoid space volume, W_BBB_: blood-brain barrier width

**
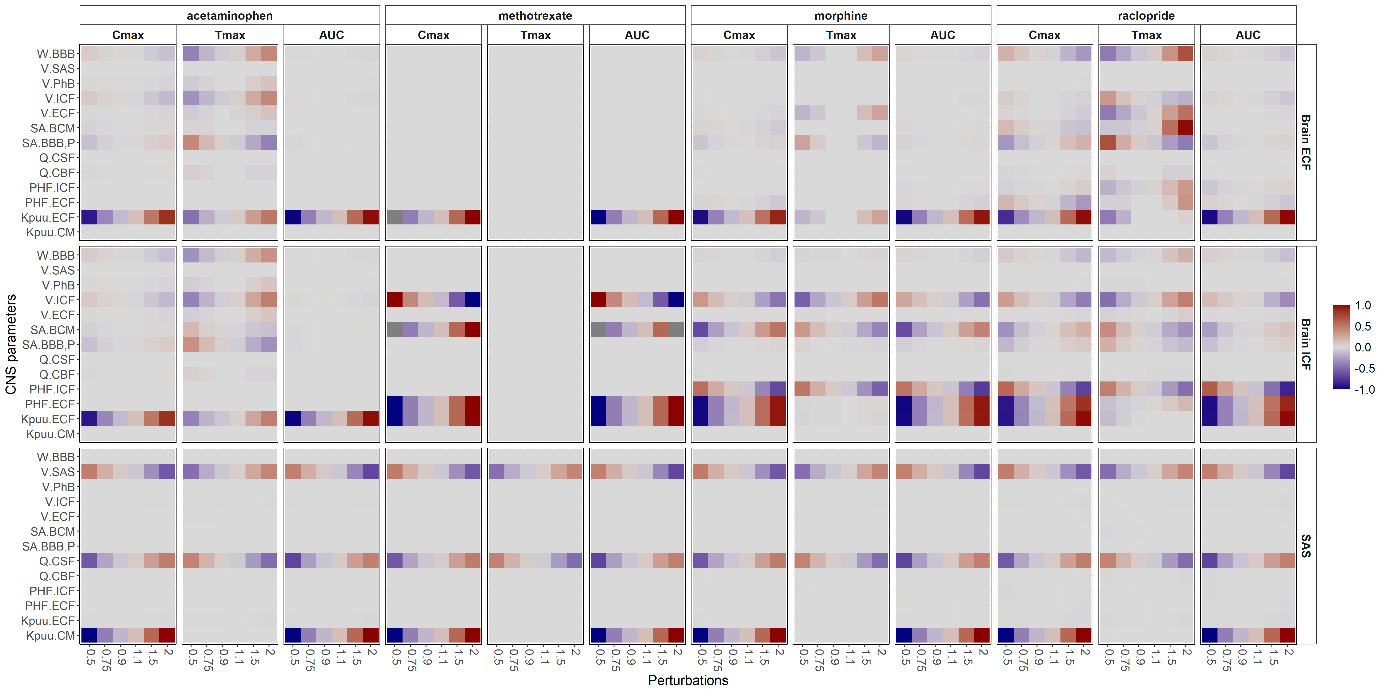
**

**Supplementary fig. 7** LeiCNS-PK3.0 simulations of acetaminophen with interindividual variability of empirical plasma model in addition to nominal variability of CNS parameters of 0%, 30%, and 50% (as %coefficient of variation). The added CNS variability results in slightly wider 2.5^th^ and 97.5^th^ percentiles that can better describe observed variability. CV: coefficient of variation (%); ECF: brain extracellular fluid; LV: lateral ventricles; CM: cisterna magna.


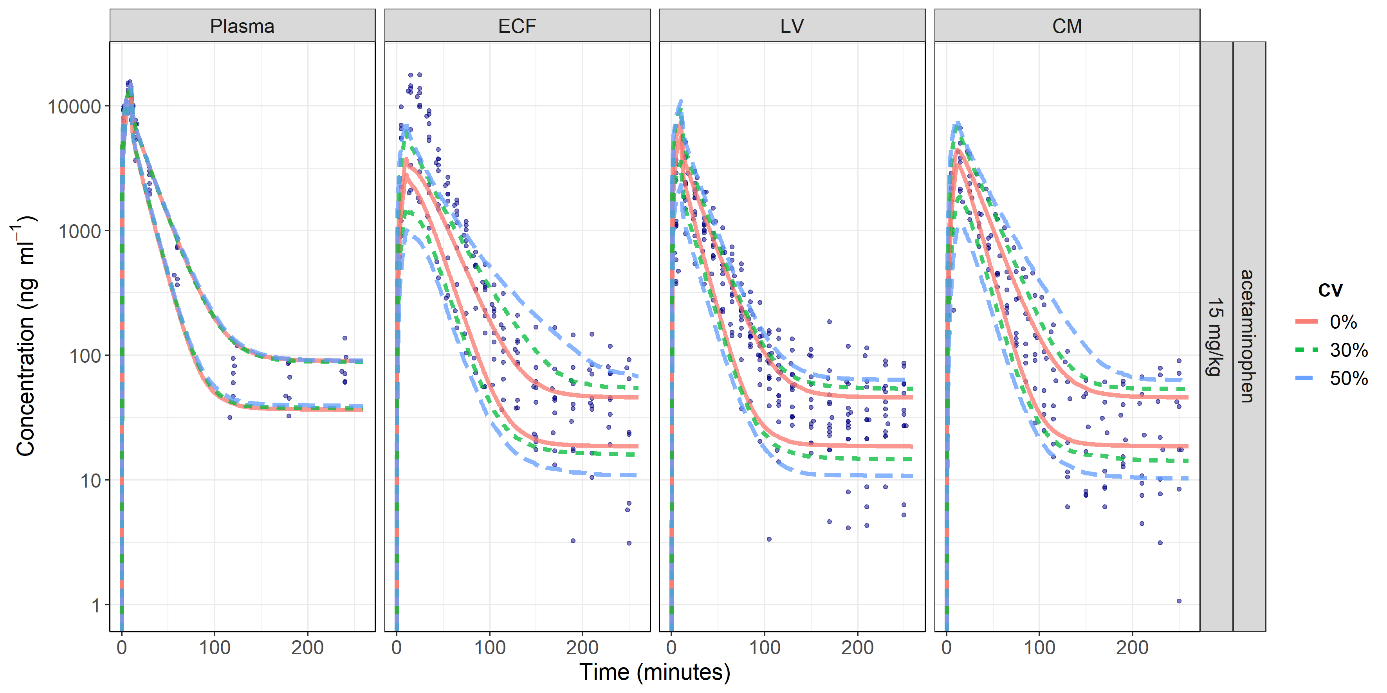


**Supplementary fig. 8** LeiCNS-PK3.0 predictions of the PK profiles of acetaminophen, indomethacin, morphine, oxycodone at plasma, brain ECF, brain ICF, and subarachnoid space. ECF: brain extracellular fluid, ICF: brain intracellular fluid, SAS: subarachnoid space.


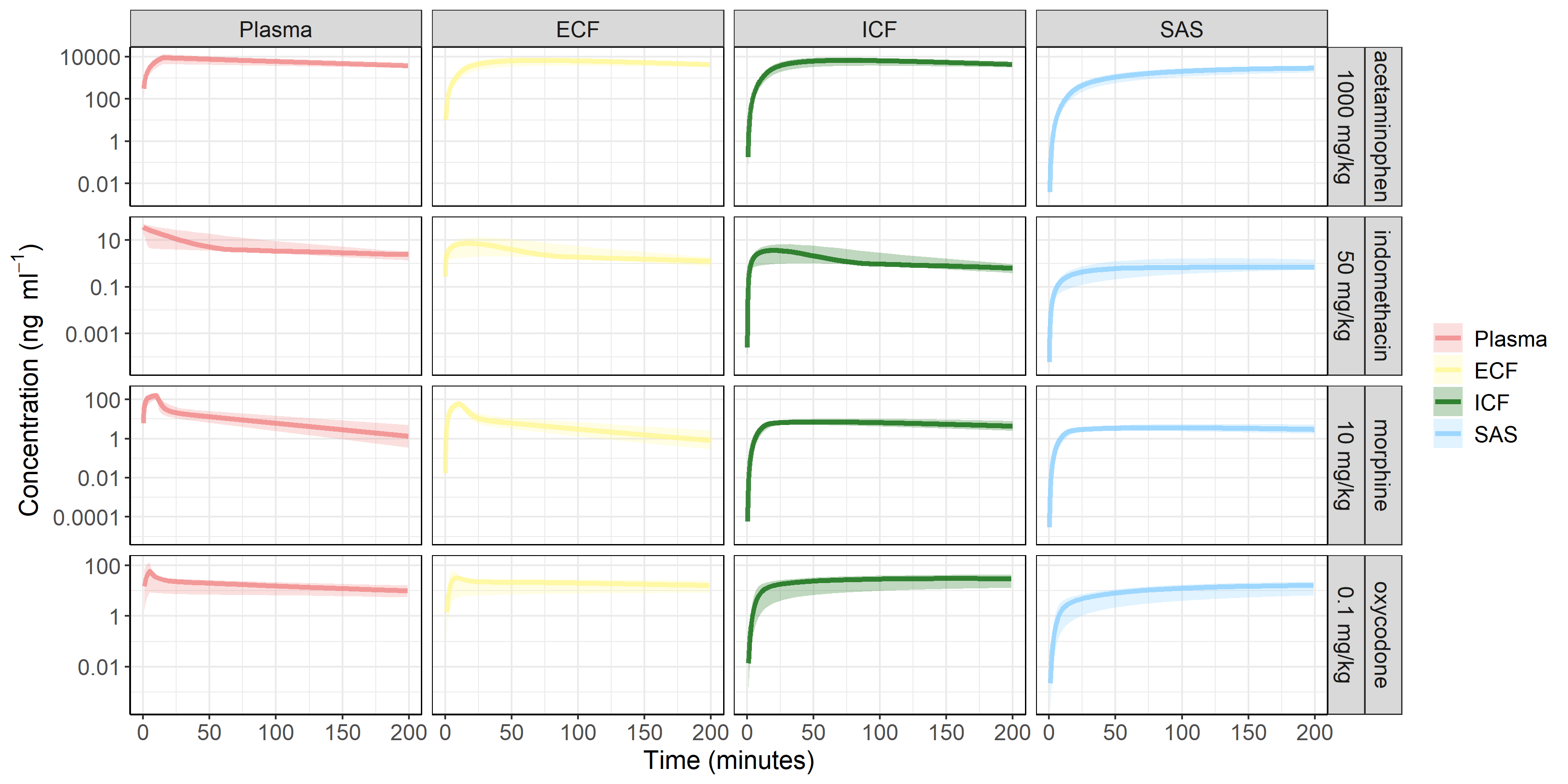


**Supplementary references**

1. Westerhout J, Ploeger B, Smeets J, et al (2012) Physiologically based pharmacokinetic modeling to investigate regional brain distribution kinetics in rats. AAPS J 14:543–553. https://doi.org/10.1208/s12248-012-9366-1

2. De Lange ECM, Danhof M, de Boer AG, Breimer DD (1994) Critical factors of intracerebral microdialysis as a technique to determined the pharmacokinetics of drugs in rat brain. Brain Res 666:1–8. https://doi.org/10.1016/0006-8993(94)90276-3

3. Westerhout J, Berg D Van Den, Hartman R, et al (2014) Prediction of methotrexate CNS distribution in different species – Influence of disease conditions. Eur J Pharm Sci 57:11–24. https://doi.org/10.1016/j.ejps.2013.12.020

4. Groenendaal D, Freijer J, De Mik D, et al (2007) Population pharmacokinetic modelling of non-linear brain distribution of morphine: Influence of active saturable influx and P-glycoprotein mediated efflux. Br J Pharmacol 151:701–712. https://doi.org/10.1038/sj.bjp.0707257

5. Bouw MR, Gårdmark M, Hammarlund-Udenaes M (2000) Modelling of morphine transport across the blood-brain barrier as a cause of the antinociceptive effect delay in rats - A microdialysis study. Pharm Res 17:1220–1227. https://doi.org/10.1023/A:1026414713509

6. Yamamoto Y, Välitalo PA, van den Berg DJ, et al (2017) A Generic Multi-Compartmental CNS Distribution Model Structure for 9 Drugs Allows Prediction of Human Brain Target Site Concentrations. Pharm Res 34:333–351. https://doi.org/10.1007/s11095-016-2065-3

7. De Lange ECMM, Westerhout J, Smeets J, et al (2013) The impact of P-gp functionality on non-steady state relationships between CSF and brain extracellular fluid. J Pharmacokinet Pharmacodyn 40:327–342. https://doi.org/10.1007/s10928-013-9314-4

8. Wong YC, Ilkova T, van Wijk RC, et al (2018) Development of a population pharmacokinetic model to predict brain distribution and dopamine D2 receptor occupancy of raclopride in non-anesthetized rat. Eur J Pharm Sci 111:514–525. https://doi.org/10.1016/j.ejps.2017.10.031

9. Yamamoto Y, Välitalo P, Huntjens D, et al (2017) Predicting drug concentration-time profiles in multiple relevant CNS compartments using a comprehensive physiologically-based pharmacokinetic model. CPT Pharmacometrics Syst Pharmacol 6:765–777. https://doi.org/10.1002/psp4.12250

10. Stevens J, Ploeger BA, Van Der Graaf PH, et al (2011) Systemic and direct nose-to-brain transport pharmacokinetic model for remoxipride after intravenous and intranasal administration. Drug Metab Dispos 39:2275–2282. https://doi.org/10.1124/dmd.111.040782

11. Bannwarth B, Netter P, Lapicque F, et al (1992) Plasma and cerebrospinal fluid concentrations of paracetamol after a single intravenous dose of propacetamol. Br J Clin Pharmacol 34:79–81. https://doi.org/10.1111/j.1365-2125.1992.tb04112.x

12. Singla NK, Parulan C, Samson R, et al (2012) Plasma and Cerebrospinal Fluid Pharmacokinetic Parameters After Single-Dose Administration of Intravenous, Oral, or Rectal Acetaminophen. Pain Pract 12:523–532. https://doi.org/10.1111/j.1533-2500.2012.00556.x

13. Bannwarth B, Netter P, Lapicque F, et al (1990) Plasma and cerebrospinal fluid concentrations of indomethacin in humans - Relationship to analgesic activity. Eur J Clin Pharmacol 38:343–346. https://doi.org/10.1007/BF00315572

14. Summerfield SG, Read K, Begley DJ, et al (2007) Central Nervous System Drug Disposition: The Relationship between in Situ Brain Permeability and Brain Free Fraction. J Pharmacol Exp Ther 322:205–213. https://doi.org/10.1124/jpet.107.121525

15. Ederoth P, Tunblad K, Bouw R, et al (2004) Blood-brain barrier transport of morphine in patients with severe brain trauma. Br J Clin Pharmacol 57:427–435. https://doi.org/10.1046/j.1365-2125.2003.02032.x

16. Kokki M, Välitalo P, Kuusisto M, et al (2014) Central nervous system penetration of oxycodone after intravenous and epidural administration. Br J Anaesth 112:133–140. https://doi.org/10.1093/bja/aet337

17. DiResta GR, Lee J, Arbit E (1991) Measurement of brain tissue specific gravity using pycnometry. J Neurosci Methods 39:245–251. https://doi.org/10.1016/0165-0270(91)90103-7

18. Kawakami J, Yamamoto K, Sawada Y, Iga T (1994) Prediction of brain delivery of ofloxacin, a new quinolone, in the human from animal data. J Pharmacokinet Biopharm 22:207–227. https://doi.org/10.1007/BF02353329

19. Courchesne E, Chisum HJ, Townsend J, et al (2000) Normal Brain Development and Aging: Quantitative Analysis at in Vivo MR Imaging in Healthy Volunteers. Radiology 216:672–682. https://doi.org/10.1148/radiology.216.3.r00au37672

20. Filipek PA, Richelme C, Kennedy DN, Caviness VS (1994) The Young Adult Human Brain: An MRI-based Morphometric Analysis. Cereb Cortex 4:344–360. https://doi.org/10.1093/cercor/4.4.344

21. Gur RC, Mozley PD, Resnick SM, et al (1991) Gender differences in age effect on brain atrophy measured by magnetic resonance imaging. Proc Natl Acad Sci U S A 88:2845–2849. https://doi.org/10.1073/pnas.88.7.2845

22. Peters M, Jäncke L, Staiger JF, et al (1998) Unsolved Problems in Comparing Brain Sizes in Homo Sapiens. Brain Cogn 37:254–285. https://doi.org/10.1006/brcg.1998.0983

23. Lei Y, Han H, Yuan F, et al (2017) The brain interstitial system: Anatomy, modeling, in vivo measurement, and applications. Prog Neurobiol 157:230–246. https://doi.org/10.1016/j.pneurobio.2015.12.007

24. Miyajima M, ARAI H (2015) Evaluation of the Production and Absorption of Cerebrospinal Fluid. Neurol Med Chir (Tokyo) 55:647–656. https://doi.org/10.2176/nmc.ra.2015-0003

25. Nicholson C (2001) Diffusion and related transport mechanisms in brain tissue. Reports Prog Phys 64:815–884. https://doi.org/10.1088/0034-4885/64/7/202

26. Nicholson C, Kamali-Zare P, Tao L (2011) Brain Extracellular Space as a Diffusion Barrier. Comput Vis Sci 14:309–325. https://doi.org/10.1007/s00791-012-0185-9

27. Thorne RG, Hrabětová S, Nicholson C (2004) Diffusion of Epidermal Growth Factor in Rat Brain Extracellular Space Measured by Integrative Optical Imaging. J Neurophysiol 92:3471–3481. https://doi.org/10.1152/jn.00352.2004

28. Weibel ER, Stäubli W, Gnägi HR, Hess FA (1969) Correlated Morphometric and Biochemical Studies on the Liver Cell: I. Morphometric Model, Stereologic Methods, and Normal Morphometric Data for Rat Liver. J Cell Biol 42:68–91. https://doi.org/10.1083/jcb.42.1.68

29. Brown RP, Delp MD, Lindstedt SL, et al (1997) Physiological parameter values for physiologically-based pharmacokinetic models. Toxicol Ind Health 13:407–484

30. Brown RP, Delp MD, Lindstedt SL, et al (1997) Physiological Parameter Values for Physiologically Based Pharmacokinetic Models. Toxicol Ind Health 13:407–484. https://doi.org/10.1177/074823379701300401

31. Hu Z-Y, Lu J, Zhao Y (2014) A physiologically based pharmacokinetic model of alvespimycin in mice and extrapolation to rats and humans. Br J Pharmacol 171:2778–2789. https://doi.org/10.1111/bph.12609

32. Preston JE (2001) Ageing choroid plexus-cerebrospinal fluid system. Microsc Res Tech 52:31–37. https://doi.org/10.1002/1097-0029(20010101)52:1<31::AID-JEMT5>3.0.CO;2-T

33. Chiu C, Miller MC, Caralopoulos IN, et al (2012) Temporal course of cerebrospinal fluid dynamics and amyloid accumulation in the aging rat brain from three to thirty months. Fluids Barriers CNS 9:1–10. https://doi.org/10.1186/2045-8118-9-3

34. Van Den Berg MP, Romeijn SG, Verhoef JC, Merkus FWHM (2002) Serial cerebrospinal fluid sampling in a rat model to study drug uptake from the nasal cavity. J Neurosci Methods 116:99–107. https://doi.org/10.1016/S0165-0270(02)00033-X

35. Basati S, Desai B, Alaraj A, et al (2012) Cerebrospinal fluid volume measurements in hydrocephalic rats. J Neurosurg Pediatr 10:347–354. https://doi.org/10.3171/2012.6.peds11457

36. Skjolding AD, Rowland IJ, Søgaard L V., et al (2010) Hydrocephalus induces dynamic spatiotemporal regulation of aquaporin-4 expression in the rat brain. Cerebrospinal Fluid Res 7:20. https://doi.org/10.1186/1743-8454-7-20

37. Levinger IM (1971) The cerebral ventricles of the rat. J Anat 108:447–451

38. Allen JS, Damasio H, Grabowski TJ (2002) Normal neuroanatomical variation in the human brain: An MRI-volumetric study. Am J Phys Anthropol 118:341–358. https://doi.org/10.1002/ajpa.10092

39. Barra V, Frenoux E, Boire J-Y (2002) Automatic volumetric measurement of lateral ventricles on magnetic resonance images with correction of partial volume effects. J Magn Reson Imaging 15:16–22. https://doi.org/10.1002/jmri.10032

40. Erdogan ALIR, DANE S, AYDIN MD, et al (2004) Sex and Handedness Differences in Size of Cerebral Ventricles of Normal Subjects. Int J Neurosci 114:67–73. https://doi.org/10.1080/00207450490249428

41. Lamers M, Klein W, Góraj B (2010) Normal values of ventricular volume and cerebrospinal fluid (CSF) circulation in healthy subjects. https://posterng.netkey.at/esr/viewing/index.php?module=viewing_poster&doi=10.1594/ecr2010/C-2729

42. Trimarchi F, Bramanti P, Marino S, et al (2013) MRI 3D lateral cerebral ventricles in living humans: morphological and morphometrical age-, gender-related preliminary study. Anat Sci Int 88:61–69. https://doi.org/10.1007/s12565-012-0162-x

43. Lebedev S V., Blinov D V., Petrov S V. (2004) Spatial characteristics of cisterna magna in rats and novel technique for puncture with a stereotactic manipulator. Bull Exp Biol Med 137:635–638. https://doi.org/10.1023/B:BEBM.0000042732.00810.01

44. Whitney N, Sun H, Pollock JM, Ross DA (2013) The human foramen magnum - Normal anatomy of the cisterna magna in adults. Neuroradiology 55:1333–1339. https://doi.org/10.1007/s00234-013-1269-z

45. Ghersi-Egea JF, Babikian A, Blondel S, Strazielle N (2015) Changes in the cerebrospinal fluid circulatory system of the developing rat: Quantitative volumetric analysis and effect on blood-CSF permeability interpretation. Fluids Barriers CNS 12:. https://doi.org/10.1186/s12987-015-0001-2

46. Conn PM (2003) Neuroscience in Medicine. Springer Science & Business Media

47. Parviz J (2011) Surgical Anatomy of the Head and Neck. Harvard University Press

48. Sakka L, Coll G, Chazal J (2011) Anatomy and physiology of cerebrospinal fluid. Eur Ann Otorhinolaryngol Head Neck Dis 128:309–316. https://doi.org/10.1016/j.anorl.2011.03.002

49. SASAKI Y, WAGNER HN (1971) Measurement of the distribution of cardiac output in unanesthetized rats. J Appl PHYSOLOGY 30:879–884

50. Lassen NA (1985) Normal Average Value of Cerebral Blood Flow in Younger Adults is 50 ml/100 g/min. J Cereb Blood Flow Metab 5:347–349. https://doi.org/10.1038/jcbfm.1985.48

51. Madsen PL, Holm S, Herning M, Lassen NA (1993) Average Blood Flow and Oxygen Uptake in the Human Brain during Resting Wakefulness: A Critical Appraisal of the Kety—Schmidt Technique. J Cereb Blood Flow Metab 13:646–655. https://doi.org/10.1038/jcbfm.1993.83

52. Pascoe MJ, Melzer TR, Horwood LJ, et al (2019) Altered grey matter volume, perfusion and white matter integrity in very low birthweight adults. NeuroImage Clin 22:101780. https://doi.org/10.1016/j.nicl.2019.101780

53. Szentistványi I, Patlak CS, Ellis RA, Cserr HF (1984) Drainage of interstitial fluid from different regions of rat brain. Am J Physiol 246:F835-844. https://doi.org/10.1152/ajprenal.1984.246.6.F835

54. Mann JD, Butler AB, Johnson RN, Bass NH (1979) Clearance of macromolecular and particulate substances from the cerebrospinal fluid system of the rat. J Neurosurg 50:343–348. https://doi.org/10.3171/jns.1979.50.3.0343

55. Abbott NJ (2004) Evidence for bulk flow of brain interstitial fluid: Significance for physiology and pathology. Neurochem Int 45:545–552. https://doi.org/10.1016/j.neuint.2003.11.006

56. Kimelberg HK (2004) Water homeostasis in the brain: Basic concepts. Neuroscience 129:851–860. https://doi.org/10.1016/j.neuroscience.2004.07.033

57. Cserr H (1965) Potassium exchange between cerebrospinal fluid, plasma, and brain. Am J Physiol 209:1219–1226. https://doi.org/10.1152/ajplegacy.1965.209.6.1219

58. Cserr HF (1971) Physiology of the choroid plexus. Physiol Rev. https://doi.org/10.1152/physrev.1971.51.2.273

59. Edsbagge M, Tisell M, Jacobsson L, Wikkelso C (2004) Spinal CSF absorption in healthy individuals. Am J Physiol - Regul Integr Comp Physiol 287:R1450–R1455. https://doi.org/10.1152/ajpregu.00215.2004

60. Lumenta CB, Rocco C Di, Haase J, Mooij JJA (2009) Neurosurgery. Springer Science & Business Media

61. Wright EM (1978) Transport processes in the formation of the cerebrospinal fluid. In: Reviews of Physiology, Biochemistry and Pharmacology, Volume 83: Volume: 83. Springer Berlin Heidelberg, Berlin, Heidelberg, pp 1–34

62. Keep RF, Jones HC (1990) A morphometric study on the development of the lateral ventricle choroid plexus, choroid plexus capillaries and ventricular ependyma in the rat. Dev Brain Res 56:47–53. https://doi.org/10.1016/0165-3806(90)90163-S

63. Sibbons PD, Aylward GL, Howard C V., Van Velzen D (1996) A quantitative immunocytochemical analysis of total surface area of blood-brain barrier in developing rat brain. Comp Haematol Int 6:214–220. https://doi.org/10.1007/BF00378113

64. Hammarlund-Udenaes M, Fridén M, Syvänen S, Gupta A (2008) On the rate and extent of drug delivery to the brain. Pharm Res 25:1737–1750. https://doi.org/10.1007/s11095-007-9502-2

65. Abbott NJ, Patabendige AAK, Dolman DEM, et al (2010) Structure and function of the blood–brain barrier. Neurobiol Dis 37:13–25. https://doi.org/10.1016/j.nbd.2009.07.030

66. Crone C (1963) The Permeability of Capillaries in Various Organs as Determined by Use of the ‘Indicator Diffusion’ Method. Acta Physiol Scand 58:292–305. https://doi.org/10.1111/j.1748-1716.1963.tb02652.x

67. Di L, Kerns EH (2015) Blood-Brain Barrier in Drug Discovery: Optimizing Brain Exposure of CNS Drugs and Minimizing Brain Side Effects for Peripheral Drugs. John Wiley & Sons

68. Gao H, Gao X (2018) Brain Targeted Drug Delivery Systems: A Focus on Nanotechnology and Nanoparticulates. Academic Press

69. Gross, Sposito, Fenstermacher (1986) Differences in Function and Structure of the Capillary Endothelium in Gray Matter, White Matter and a Circumventricular Organ of Rat Brain - Abstract - Journal of Vascular Research 1986, Vol. 23, No. 6 - Karger Publishers. https://www.karger.com/Article/Abstract/158652

70. Pardridge WM (2005) The Blood-Brain Barrier: Bottleneck in Brain Drug Development. NeuroRx 2:3–14. https://doi.org/10.1602/neurorx.2.1.3

71. Redzic Z (2011) Molecular biology of the blood-brain and the blood-cerebrospinal fluid barriers: similarities and differences. Fluids Barriers CNS 8:3. https://doi.org/10.1186/2045-8118-8-3

72. Sharma HS (2003) Blood-Spinal Cord and Brain Barriers in Health and Disease. Elsevier

73. Wong AD, Ye M, Levy AF, et al (2013) The blood-brain barrier: an engineering perspective. Front Neuroeng 6:1–22. https://doi.org/10.3389/fneng.2013.00007

74. Pardridge WM (2016) CSF, blood-brain barrier, and brain drug delivery. Expert Opin Drug Deliv 13:963–975. https://doi.org/10.1517/17425247.2016.1171315

75. Spector R, Keep RF, Robert Snodgrass S, et al (2015) A balanced view of choroid plexus structure and function: Focus on adult humans. Exp Neurol 267:78–86. https://doi.org/10.1016/j.expneurol.2015.02.032

76. Herculano-Houzel S, Mota B, Lent R (2006) Cellular scaling rules for rodent brains. Proc Natl Acad Sci 103:12138–12143. https://doi.org/10.1073/pnas.0604911103

77. Andrade-Moraes CH, Oliveira-Pinto A V, Castro-Fonseca E, et al (2013) Cell number changes in Alzheimer’s disease relate to dementia, not to plaques and tangles. Brain 136:3738–3752. https://doi.org/10.1093/brain/awt273

78. Azevedo FAC, Carvalho LRB, Grinberg LT, et al (2009) Equal numbers of neuronal and nonneuronal cells make the human brain an isometrically scaled-up primate brain. J Comp Neurol 513:532–541. https://doi.org/10.1002/cne.21974

79. Bakker AC, Webster P, Jacob WA, Andrews NW (1997) Homotypic fusion between aggregated lysosomes triggered by elevated [Ca2+]i in fibroblasts. J Cell Sci 110:2227–38

80. Bandyopadhyay D, Cyphersmith A, Zapata JA, et al (2014) Lysosome transport as a function of lysosome diameter. PLoS One 9:e86847. https://doi.org/10.1371/journal.pone.0086847

81. Demers-Lamarche J, Guillebaud G, Tlili M, et al (2016) Loss of Mitochondrial Function Impairs Lysosomes. J Biol Chem 291:10263–10276. https://doi.org/10.1074/jbc.M115.695825

82. RAHMAN YE (1962) Electron microscopy of lysosome-rich fractions from rat thymus isolated by density-gradient centrifugation before and after whole-body x-irradiation. J Cell Biol 13:253–260. https://doi.org/10.1083/jcb.13.2.253

83. Xu H, Ren D (2015) Lysosomal Physiology. Annu Rev Physiol 77:57–80. https://doi.org/10.1146/annurev-physiol-021014-071649

84. Keep RF, Jones HC (1990) Cortical microvessels during brain development: A morphometric study in the rat. Microvasc Res 40:412–426. https://doi.org/10.1016/0026-2862(90)90036-Q

85. Cornford EM, Hyman S, Cornford ME, et al (1998) Interictal Seizure Resections Show Two Configurations of Endothelial Glut1 Glucose Transporter in the Human Blood–Brain Barrier. J Cereb Blood Flow Metab 18:26–42. https://doi.org/10.1097/00004647-199801000-00003

86. Sarin H (2010) Physiologic upper limits of pore size of different blood capillary types and another perspective on the dual pore theory of microvascular permeability. J Angiogenes Res 2:14. https://doi.org/10.1186/2040-2384-2-14

87. Monteiro JN, Goraksha SU (2017) ‘ROSE concept’ of fluid management: Relevance in neuroanaesthesia and neurocritical care. J Neuroanaesth Crit Care 04:10–16. https://doi.org/10.4103/2348-0548.197435

88. Haas TL, Duling BR (1997) Morphology favors an endothelial cell pathway for longitudinal conduction within arterioles. Microvasc Res 53:113–120. https://doi.org/10.1006/mvre.1996.1999

89. Schulze C, Firth JA (1992) Interendothelial junctions during blood-brain barrier development in the rat: morphological changes at the level of individual tight junctional contacts. Dev Brain Res 69:85–95. https://doi.org/10.1016/0165-3806(92)90125-G

90. Atherton JC (2003) Acid-base balance: maintenance of plasma pH. Anaesth Intensive Care Med 4:419–422. https://doi.org/10.1383/anes.4.12.419.27385

91. Fridén M, Bergström F, Wan H, et al (2011) Measurement of unbound drug exposure in brain: modeling of pH partitioning explains diverging results between the brain slice and brain homogenate methods. Drug Metab Dispos 39:353–362. https://doi.org/10.1124/dmd.110.035998

92. Siesjö BK (1972) Symposium on acid-base homeostasis. The regulation of cerebrospinal fluid pH. Kidney Int 1:360–374. https://doi.org/10.1038/ki.1972.47

93. Weiss N, Miller F, Cazaubon S, Couraud PO (2009) The blood-brain barrier in brain homeostasis and neurological diseases. Biochim Biophys Acta - Biomembr 1788:842–857. https://doi.org/10.1016/j.bbamem.2008.10.022

1. Based on rat brain weight (1.88 gm) and density (1.04-1.05 gm ml**^-1^**) [↑](#footnote-ref-1)
2. Calculated as 15-20 (20 was used)% of total brain volume [↑](#footnote-ref-2)
3. Calculated as 80% of total brain volume [↑](#footnote-ref-3)
4. Calculated as 1.25% (1/80) of ICF volume; based on liver lysosomes [↑](#footnote-ref-4)
5. Calculated as 3% of total brain volume [↑](#footnote-ref-5)
6. Calculated as 3.67% of total brain volume [↑](#footnote-ref-6)
7. Mean of the 4 values [↑](#footnote-ref-7)
8. Assuming equal volumes of the ventricles; based on volumes of three-month-old rats [↑](#footnote-ref-8)
9. [Calculated as 5.7% of total CSF volume and according to cisterna magna geometry](https://www.ncbi.nlm.nih.gov/pmc/articles/PMC4365764/) [↑](#footnote-ref-9)
10. Calculated as 48% of total CSF volume, based on measurement performed in 9-day-old rats [↑](#footnote-ref-10)
11. Calculated as 2.6% of total cardiac output [↑](#footnote-ref-11)
12. Based on 50% of CSF bulk flow [↑](#footnote-ref-12)
13. Based on three-month-old rats, surface area at lateral ventricles (and 3^rd^ and 4^th^ ventricles) is assumed 50% of total surface area [↑](#footnote-ref-13)
14. Based on 0.1 of BBB surface area, surface area at lateral ventricles (and 3^rd^ and 4^th^ ventricles) is assumed 50% of total surface area [↑](#footnote-ref-14)
15. Based on ICF total volume, total number of brain cells, (1)and assuming spherical cells to calculate the radius which is used with total number of brain cells to calculate total surface area of brain cell membranes [↑](#footnote-ref-15)
16. Based on lysosomes total volume and the average radius of rat kidney lysosomes (0.2 µm) [↑](#footnote-ref-16)
17. Based on lysosomes total volume and the average radius of monkey kidney and rat kidney lysosomes (0.1875 µm) [↑](#footnote-ref-17)
18. Based on 1500 gm brain [↑](#footnote-ref-18)
19. Based on relative length of intercellular space (0.03 μm) and cell perimeter (17 μm) [9, 93] [↑](#footnote-ref-19)
20. Assumed the same as rats [↑](#footnote-ref-20)
21. Based on an endothelial cell perimeter of 17 um [↑](#footnote-ref-21)
